# Comparing the treatment effects of online cognitive-behavioral therapy for pediatric functional abdominal pain disorders with and without psychiatric comorbidity

**DOI:** 10.1177/17562848251384605

**Published:** 2025-10-09

**Authors:** Viktor Vadenmark Lundqvist, Aleksandra Bujacz, Jenny Rickardsson, Johan Åhlén, Martin Jonsjö, Jörgen Rosén, Sarah Vigerland, Karin Jensen, Marianne Bonnert, Maria Lalouni

**Affiliations:** Department of Clinical Neuroscience, Karolinska Institutet, Nobels Väg 9, Stockholm 171 65, Sweden; Department of Learning, Informatics, Management and Ethics, Karolinska Institutet, Stockholm, Sweden; Department of Clinical Neuroscience, Karolinska Institutet, Stockholm, Sweden; Center for Epidemiology and Community Medicine, Region Stockholm, Stockholm, Sweden; Department of Global Public Health, Karolinska Institutet, Stockholm, Sweden; Department of Clinical Neuroscience, Karolinska Institutet, Stockholm, Sweden; Department of Physical Activity and Health, The Swedish School of Sport and Health Sciences, Stockholm, Sweden; Medical Psychology, Women’s Health and Allied Health Professionals Theme, Karolinska University Hospital, Solna, Sweden; Department of Clinical Neuroscience, Karolinska Institutet, Stockholm, Sweden; Department of Clinical Neuroscience, Karolinska Institutet, Stockholm, Sweden; Department of Clinical Neuroscience, Karolinska Institutet, Stockholm, Sweden; Department of Clinical Neuroscience, Karolinska Institutet, Stockholm, Sweden; Department of Clinical Neuroscience, Karolinska Institutet, Stockholm, Sweden; Center for Epidemiology and Community Medicine, Region Stockholm, Stockholm, Sweden

**Keywords:** cognitive behavioral therapy, functional abdominal pain, IBS, internet-delivered CBT, psychiatric comorbidity

## Abstract

**Background:**

Functional abdominal pain disorders (FAPDs) are disorders of the gut-brain interaction. FAPDs are common in children and adolescents (global prevalence 12%) and are associated with psychiatric comorbidity. Internet-delivered cognitive-behavioral therapy (iCBT) is effective for FAPDs, but it’s unclear whether children with psychiatric comorbidities benefit equally from the treatment.

**Objectives:**

In this study, we assessed whether having a comorbid psychiatric diagnosis results in different rates of change in iCBT for children with FAPDs.

**Design:**

Between-groups design.

**Methods:**

Participants were 120 children with FAPDs (age 8–12 years) taking part in one of two clinical trials testing 10 weeks of iCBT. For the analyses, participants were divided into groups: presence or absence of psychiatric disorder. The primary outcome was gastrointestinal symptoms, assessed weekly using the Pediatric Quality of Life Gastrointestinal Symptom Scale. Secondary outcomes included health-related quality of life, gastrointestinal-specific anxiety, and pain intensity. Multilevel modeling was used to assess differences in rates of change between groups from baseline to follow-up directly after treatment, and then to 6-month follow-up.

**Results:**

We observed significant improvements in the rates of change for both groups for the primary outcome (gastrointestinal symptoms) and all secondary outcomes during treatment. Children with psychiatric comorbidity had significantly more severe symptoms at baseline on all measures, but there was no difference in the rates of change for the primary outcome (−0.29, 95% confidence interval (CI): −0.70, 0.11, *p* = 0.159) or any of the secondary outcomes compared to the non-comorbid group. Treatment benefits were sustained at 6-month follow-up.

**Conclusion:**

ICBT seems to be beneficial for children with FAPDs, also in the presence of psychiatric comorbidity. Given the high prevalence of psychiatric comorbidity in this patient group, the results will aid the clinical assessment and treatment planning for these patients.

## Plain language summary

Comparing internet treatment for children with functional abdominal pain, with and without psychiatric disorders

This study looked at how well internet-delivered cognitive behavioral therapy (iCBT) works for children with functional abdominal pain disorders (FAPDs), both with and without psychiatric conditions like anxiety or depression. FAPDs are common in children and can cause significant discomfort and reduced quality of life. Many children with FAPDs also have psychiatric conditions. The study included 120 children aged 8-12 years who previously received 10 weeks of iCBT. The children were divided into two groups: those with psychiatric conditions and those without. The results showed that iCBT helped reduce gastrointestinal symptoms and improve quality of life for all children, regardless of whether they had a psychiatric condition. Children with psychiatric conditions started with more severe symptoms but improved at the same rate as those without psychiatric conditions. The benefits of iCBT were sustained at follow-up six months after treatment. This study suggests that iCBT is an effective treatment for children with FAPDs, even if they have psychiatric conditions. This is important because many children with FAPDs have psychiatric conditions, and this treatment can help improve their overall well-being.

## Introduction

Pain-related disorders of gut-brain interaction, also called functional abdominal pain disorders (FAPDs), are a group of gastrointestinal conditions without an identifiable structural or biochemical cause.^1^ Instead, these conditions are marked by changes in gut-brain axis interaction and regulation of visceral pain.^2^ FAPDs involve recurring or persistent abdominal pain along with other gastrointestinal symptoms, such as bloating. FAPDs include irritable bowel syndrome, functional dyspepsia, abdominal migraine, and functional abdominal pain—not otherwise specified.

FAPDs are common, with a global prevalence of about 12% in children and adolescents,^3^ and are associated with significant suffering and high societal costs.^4,5^ Although FAPDs are not associated with organic abnormalities, they can lead to significant impairment and reduced quality of life for affected children.^6^

There is a notable overlap between abdominal pain and psychiatric challenges, particularly in children and adolescents. In child and adolescent psychiatry, recurring abdominal pain is more common than in the general population,^7^ and research indicates that around 20%–50% of children with FAPDs have comorbid anxiety or depression.^8^ Children diagnosed with FAPDs exhibit an elevated risk of psychiatric challenges, a risk that persists even after the resolution of abdominal pain.^9^ Even solely the presence of abdominal symptoms in adolescents may serve as a significant predictor of severe mental health disorders in adulthood.^10^ This relationship is likely bidirectional, meaning that psychiatric issues may contribute to the development of FAPDs, which in turn can lead to psychiatric vulnerability.^11,12^ For example, anxiety and depression early in life have been shown to increase the risk of developing FAPD later in life.^13^

Such findings align with the predominant biopsychosocial model of FAPDs, which posits that the development, persistence, and impact of these disorders cannot be attributed solely to biological mechanisms. Instead, they are shaped by the dynamic interaction of physiological processes, emotional and cognitive states, and social and environmental influences.^11,12^ Research on the gut-brain axis has underscored the intricate and bidirectional relationship between gastrointestinal and psychological factors. For instance, it has been demonstrated that the gut microbiota plays a pivotal role in regulating stress responses and stress-related behaviors,^14^ influencing both emotional and physiological outcomes. In addition, experimentally induced stress has been shown to alter visceral perception in patients with irritable bowel syndrome, further illustrating the dynamic interplay between psychological stressors and gastrointestinal function.^15^ Such findings highlight the biopsychosocial nature of FAPDs and that psychological factors, such as depression and anxiety, might interfere with abdominal symptoms.

Having a comorbid psychiatric diagnosis may negatively impact treatment response in chronic pain conditions. A recent meta-analysis, including 23 studies, concluded that adults with functional pain and related conditions (e.g., irritable bowel syndrome) and comorbid anxiety or depression had a less favorable treatment outcome after CBT.^16^ Similar findings have also been observed in children. Psychiatric comorbidity in children with chronic pain, and FAPDs specifically, has been shown to exacerbate symptoms, negatively impact treatment response, and increase disability.^17–20^ There is, however, still a scarcity of studies investigating the influence of comorbid psychopathology on treatment effect in the pediatric pain population.

Pediatric chronic pain can be challenging to treat and often persists into adulthood.^21^ This indicates that the need for effective treatment is urgent. Recent studies from our group have shown that internet-delivered cognitive-behavioral therapy (iCBT) is an effective treatment for FAPDs.^22,23^ Children who received iCBT had a significantly greater improvement in gastrointestinal symptoms, quality of life, gastrointestinal-specific anxiety, and gastrointestinal-related avoidance behaviors compared to a treatment-as-usual group.^23^ Although the improvements were significant and persisted through 36 weeks of follow-up, it remains unclear whether iCBT is equally effective for children with and without comorbid psychiatric problems.

The present study aimed to explore whether psychiatric comorbidity affects the outcomes of iCBT for FAPDs in children. Based on previous studies in adult populations, we hypothesized that those with a psychiatric disorder would benefit less from the treatment than those without a comorbid disorder. To the best of our knowledge, this is the first study to investigate the differential effects of comorbid psychopathology on internet-delivered, exposure-based CBT in young patients with FAPDs.

## Materials and methods

### Participants

This study used secondary data pooled from two previous clinical trials, a feasibility study (*n* = 31)^24^ and a randomized controlled trial (RCT, *n* = 89).^23^ In both studies, children aged 8–12 years with FAPDs received 10 weeks of iCBT with the aim of reducing their gastrointestinal symptoms. Participants were referred to the studies by their physicians, who certified the FAPD diagnosis.

Children from across Sweden participated in the studies at the Child and Adolescent Psychiatry Research Center in Stockholm, between August 2015 and January 2016 for the feasibility study, and between September 2016 and April 2017 for the RCT. In the feasibility study, all children received Internet-CBT. In the RCT, 46 children were randomized to receive immediate iCBT, while 45 were assigned to treatment as usual and crossed over to iCBT after 10 weeks (iCBT-delayed). One child was removed from each group: from the iCBT group due to unreliable questionnaire responses and from the iCBT-delayed group due to a new somatic disorder diagnosis. Thus, the current study sample includes 120 children who all received iCBT.

Inclusion criteria for the studies were: (a) ⩾8 and ⩽12 years of age, (b) fulfilling the ROME criteria (feasibility study: ROME III, RCT: ROME IV) for at least one of the following FAPDs: irritable bowel syndrome, functional dyspepsia, or functional abdominal pain not otherwise specified, (c) stable dose for at least a month if psychopharmacological medications were used, (d) internet access, and (e) basic writing and reading skills (the child and at least one parent). The exclusion criteria were: (f) other disease that could better explain the abdominal symptoms; (g) severe psychiatric or social problems in need of immediate other care (decided in collaboration with a child and adolescent psychiatrist); (h) school absence >40%; and (i) other ongoing psychological treatment.

### Psychiatric assessment

At inclusion in the study, all subjects underwent a structured psychiatric interview at the clinic by a clinical psychologist, including the Mini-International Neuropsychiatric Interview for Children and Adolescents (MINI-KID^25^). MINI-KID assesses the presence of psychiatric disorders according to the DSM-IV and ICD-10 diagnostic manuals in children 6–17 years. It is administered to the child with the parent present. MINI-KID has shown very good to excellent test-retest reliability, high interrater reliability, and substantial to excellent criterion validity for detecting common psychiatric disorders.^25^ Information was also collected from parents regarding whether their children had any current neurodevelopmental disorder (autism spectrum disorder or attention deficit hyperactivity disorder) diagnosed elsewhere.

### Internet-delivered cognitive behavioral therapy

The iCBT consisted of 10 weekly modules for children as well as 10 weekly modules for their respective parents. The main component in the child modules was exposure to pain-provoking stimuli (e.g., foods or physical activity) and having abdominal symptoms in feared situations (e.g., being in school or attending social activities). The aim of the parental modules was to help parents support their child’s exposure exercises and redirect parental attention away from the child’s illness behaviors toward other important areas of the child’s life (e.g., school, play, leisure activities). For a more thorough treatment description, see Lalouni et al.^24,26^

### Procedure

**Figure 1.**
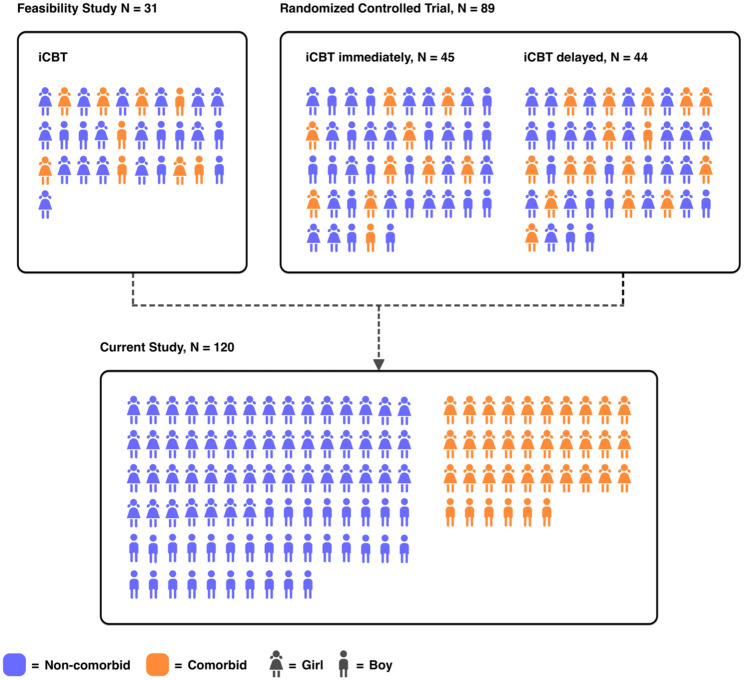
A diagram of how data from the two original studies were combined in the present study, dividing children into two new groups: absence (non-comorbid) or presence (comorbid) of psychiatric comorbidity.

In the present study, the sample consisted of 120 participants. For the analyses, the participants were divided into two groups: (1) presence (*n* = 36) or (2) absence (*n* = 84) of any psychiatric disorder (Figure 1).

### Outcome measures

All outcome measures were self-reported by the children and assessed via an internet platform from the participants’ homes, without the influence of the researchers. Only outcome measures collected in both studies were included in the present analysis.

#### Primary outcome

The primary outcome was gastrointestinal symptoms, assessed using the Pediatric Quality of Life Gastrointestinal Symptom Scale (PedsQL Gastro).^27^ PedsQL Gastro is a scale of 0–100, where higher scores indicate lower symptom severity.

PedsQL Gastro was assessed weekly during the 10-week treatment period using a 1-week recall period. In the RCT, the children randomized to treatment as usual were assessed biweekly once they had crossed over to iCBT (the period of interest for the present study). At the follow-up, 6 months after treatment completion, a 1-month recall period was used.

#### Secondary outcomes

Health-related quality of life was assessed using the Pediatric Quality of Life Inventory (PedsQL QOL).^28^ PedsQL QOL is a scale of 0–100, where higher scores indicate a higher quality of life.

Gastrointestinal-specific anxiety was assessed by the child using the Visceral Sensitivity Index—Child-adapted version (VSI-C).^29^ VSI-C is a scale of 0–35, where lower scores indicate lower symptom severity.

Last week’s worst pain intensity was assessed by the child using the Faces Pain Rating Scale (FACES).^30^ FACES is a scale of 0–10, where lower scores indicate lower pain intensity.

Anxiety symptoms were assessed using the Spence Children’s Anxiety Scale—Short version (SCAS-S).^31^ SCAS-S is a scale of 0–54, where lower scores indicate lower symptom severity.

Depressive symptoms were assessed using the Child Depression Inventory—Short version (CDI-S).^32^ CDI-S is a scale of 0–20, where lower scores indicate lower symptom severity.

All secondary measures were assessed at baseline, at follow-up after iCBT, and at follow-up 6 months after treatment completion.

### Statistical analyses

For the primary outcome, multilevel modeling was used to assess differences in rates of change (growth) in gastrointestinal symptoms for children with and without psychiatric comorbidity from baseline to follow-up after treatment, including weekly/biweekly measurements. All available data were used to model rates of change. A description of missing values, handled within the multilevel model, is available in Supplemental Material 1. We also tested differences between groups directly after the treatment and at the 6-month follow-up using a multilevel model.

For the secondary outcomes, multilevel models were used to assess rates of change from baseline to follow-up directly after treatment, and from after the treatment to the 6-month follow-up.

The models estimated three fixed effects for each outcome (the difference in the group with comorbidities when compared to the group without comorbidities; the change over time for the group without comorbidities; and the time-by-group interaction, which is also the same as the average impact of comorbidities on treatment effects in the study population) and a random residual (participant-level deviations around the baseline grand mean). Each model includes two rates of change effects and two interaction effects: (1) for the period between baseline and follow-up immediately after treatment, and (2) between the follow-up immediately after treatment to the 6-month follow-up. The analyses were conducted using the GAMLj module^33^ in jamovi (version 2.2),^34^ running R (Version 4.0).^35^

## Results

In total, 210 children were referred to the original studies by their physicians, of which 60 were excluded and 30 declined to participate. Reasons for exclusion are described in detail in the original articles.^23,24^ The most common reason for exclusion was that the child did not fulfill criteria for a FAPD diagnosis (*n* = 35). Only four children were excluded because of severe psychiatric or social problems in need of other treatment, and two children were excluded because they had >40% school absenteeism. Out of 120 children included in the study, 36 (30%) had at least one psychiatric comorbid diagnosis. Children with psychiatric comorbidity had significantly worse baseline levels of all assessed outcome measures compared to children without psychiatric comorbidity. See Table 1 for baseline demography, Supplemental Material 1 for descriptive statistics, and Supplemental Material 4 for observed means and standard deviations at all time points.

**Table 1.** Baseline demographics and clinical characteristics.

Demographics and characteristics	Total (*n* = 120)	Comorbid (*n* = 36, 30%)	Non-comorbid (*n* = 84, 70%)	
Age, *M* (SD)	10.4 (1.3)	10.4 (1.3)	10.4 (1.3)	
Female, *n* (%)	81 (67.5)	29 (80.6)	52 (61.9)	
Duration of abdominal symptoms, years (SD)	3.7 (2.3)	3.6 (2.1)	3.7 (2.3)	
Parents and child born in Sweden, *n* (%)	95 (79)	30 (83)	65 (77)	
Child’s living arrangement				
Both parents (cohabiting), *n* (%)	94 (78)	30 (83)	64 (76)	
Both parents (separate homes), *n* (%)	14 (12)	4 (11)	10 (12)	
One parent, *n* (%)	12 (10)	2 (6)	10 (12)	
Parental characteristics^a^				
Mothers, *n* (%)	104 (87)	33 (92)	71 (85)	
Parents’ highest education				
Elementary school, *n* (%)	3 (3)	1 (3)	2 (2)	
High school, *n* (%)	25 (21)	8 (22)	17 (20)	
College/university, *n* (%)	89 (74)	25 (69)	64 (76)	
Other, *n* (%)	3 (3)	2 (6)	1 (1)	
Baseline symptoms				*p* Diff
PedsQL Gastro^b^, *M* (SD)	61.7 (13.8)	57.7 (15.3)	63.2 (13.0)	0.025
Peds QL QoL^b^, *M* (SD)	75.8 (12.9)	69.5 (14.9)	78.2 (11.6)	<0.001
VSI, *M* (SD)	11.6 (7.8)	14.0 (9.2)	10.7 (7.0)	0.016
FACES, *M* (SD)	6.1 (2.3)	6.8 (1.9)	5.8 (2.4)	0.012
SCAS, *M* (SD)	13.0 (7.9)	17.8 (9.7)	11.3 (7.4)	<0.001
CDI, *M* (SD)	2.9 (2.8)	4.3 (3.6)	2.3 (2.2)	<0.001

a Parental characteristics refer to the parent responsible for assessments and treatment.

b PedsQL Gastro and Peds QL QoL are inversely rated, with higher values indicating fewer symptoms and better health.

CDI-S, Child Depression Inventory—Short version; FACES, Faces Pain Rating Scale; PedsQL Gastro, Pediatric Quality of Life Gastrointestinal Symptom Scale; Peds QL QoL, Pediatric Quality of Life Inventory; SCAS-S, Spence Children Anxiety Scale—Short version; VSI, Visceral Sensitivity Index.

The children with psychiatric comorbidities had between one and four coexisting conditions. Anxiety disorders were most prevalent, affecting 27 children (75%). The anxiety disorders were specific phobia (*n* = 20), separation anxiety (*n* = 8), generalized anxiety disorder (*n* = 6), social anxiety disorder (*n* = 3), and agoraphobia (*n* = 1). Depressive disorders were present in 10 children (28%) and consisted of major depressive disorder (*n* = 6), depression not otherwise specified (*n* = 3) and persistent depressive disorder (*n* = 1), while obsessive compulsive disorder, tics, or other disorders were observed in seven children (19%). Two children in the psychiatric comorbidity group had pharmacological medication at baseline (fluoxetine and atomoxetine). Both these children had stopped taking their medication at the follow-up assessments.

### Primary outcome

**Figure 2.**
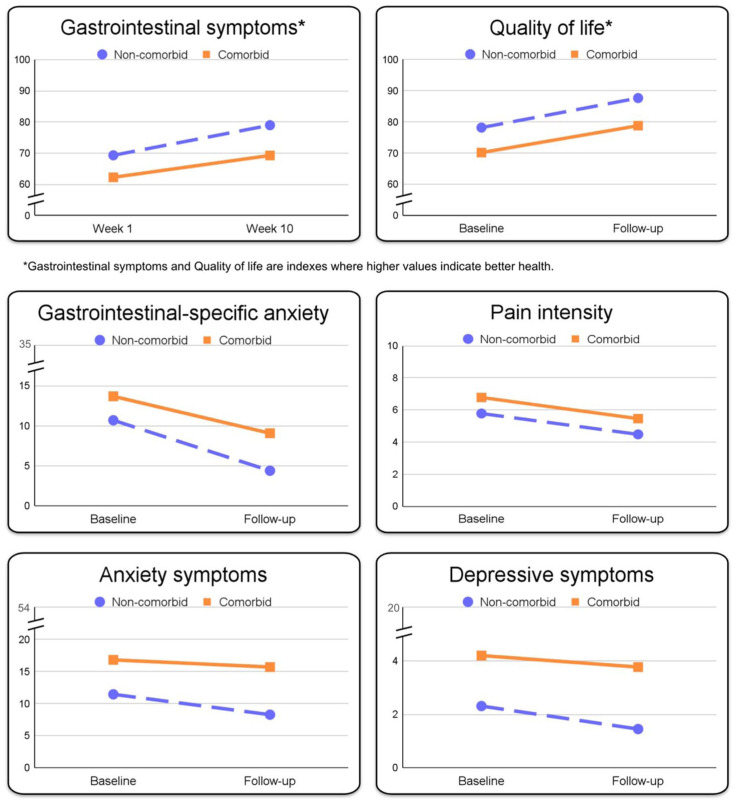
Estimated mean change slopes during treatment for participants with (*n* = 36) and without (*n* = 84) psychiatric comorbidity. Gastrointestinal symptoms and quality of life were measured with PedsQL. Gastrointestinal-specific anxiety was measured with the Visceral Sensitivity Index, Pain intensity with the Faces Pain Rating Scale, Anxiety symptoms with the Spence Children Anxiety scale, and depressive symptoms with the Child Depression Inventory. PedsQL, Pediatric Quality of Life.

In the model, the group without psychiatric comorbidity served as the reference group, and the difference in rate of change indicates how the group with psychiatric comorbidity performed relative to this reference group. Between baseline to follow-up immediately after treatment, the rate of improvement of gastrointestinal symptoms was on average 1.07 (95% confidence interval (CI): 0.85, 1.29, *p* < 0.001) per week for the group without psychiatric comorbidity (Figure 2). No significant difference between the groups in weekly rates of change was observed (−0.29, 95% CI: −0.70, 0.11, *p* = 0.159). Model info, fixed effect omnibus test, and fixed effects parameter estimates are presented in Tables 2–4. A complete description of the analysis is presented in Supplemental Material 2.

**Table 2.** Model info.

Model attributes	Values
Estimate	Linear mixed model fit by REML
Call	Gastro ~ 1 + ttreat + comorb + tfollow + ttreat:comorb + comorb:tfollow + (1 + ttreat|id)
AIC	8998.067
BIC	9043.148
LogLikel.	−4485.996
*R*-squared marginal	0.104
*R*-squared conditional	0.772
Converged	Yes
Optimizer	Bobyqa

AIC, Akaike information criterion; BIC, Bayesian information criterion; REML, restricted maximum likelihood.

**Table 3.** Fixed effects omnibus tests.

Names	*F*	Num df	Den df	*p*
ttreat	79.496	1	134	<0.001
comorb	9.985	1	123	0.002
tfollow	0.387	1	1015	0.534
ttreat * comorb	2.005	1	134	0.159
comorb * tfollow	1.197	1	1015	0.274

Satterthwaite method for degrees of freedom. ttreat = weekly rate of change for the non-comorbid group during treatment; comorb1 = difference in intercept for the comorbid group; tfollow = rate of change between the end of treatment and 6-month follow-up for the non-comorbid group; ttreat * comorb1 = difference in rate of change between the two groups during treatment; comorb1 * tfollow = difference in rate of change between the two groups between the end of treatment and 6-month follow-up.

**Table 4.** Fixed effects parameter estimates.

Names	Effect	Estimate	SE	95% confidence interval		df	*t*	*p*
				Lower	Upper			
(Intercept)	(Intercept)	78.979	1.668	75.711	82.247	122	47.36	<0.001
ttreat	ttreat	1.073	0.112	0.853	1.293	130	9.56	<0.001
comorb1	1–0	−9.710	3.073	−15.733	−3.687	123	−3.16	0.002
tfollow	tfollow	−1.627	1.022	−3.630	0.376	1013	−1.59	0.112
ttreat * comorb1	ttreat * 1–0	−0.294	0.208	−0.701	0.113	134	−1.42	0.159
comorb1 * tfollow	1–0 * tfollow	2.074	1.896	−1.642	5.791	1015	1.09	0.274

ttreat = weekly rate of change for the non-comorbid group during treatment; comorb1 = difference in intercept for the comorbid group; tfollow = rate of change between the end of treatment and 6-month follow-up for the non-comorbid group; ttreat * comorb1 = difference in rate of change between the two groups during treatment; comorb1 * tfollow = difference in rate of change between the two groups between the end of treatment and 6-month follow-up.

df, degrees of freedom; SE, standard error.

The rates of change for the group without psychiatric comorbidity between the follow-up immediately after treatment and the 6-month follow-up were nonsignificant (−1.63, 95% CI: −3.63, 0.38, *p* = 0.112). There was no significant difference between groups in rate of change (2.07, 95% CI: −1.64, 5.79, *p* = 0.274).

### Secondary outcomes

All secondary outcomes showed significant rates of improvement from baseline to follow-up immediately after treatment for the group without psychiatric comorbidity: health-related quality of life 9.47 (95% CI: 7.02, 11.91, *p* < 0.001); gastrointestinal-specific anxiety −6.30 (95% CI: −7.70, −4.89, *p* < 0.001); last week’s worst pain intensity −1.30 (95% CI: −1.91, −0.69, *p* < 0.001); anxiety symptoms −3.18 (95% CI: −4.43, −1.94, *p* < 0.001); and depressive symptoms −0.86 (95% CI: −1.34, −0.39, *p* < 0.001).

No significant differences between groups with and without psychiatric comorbidity in rate of change between baseline and follow-up immediately after treatment were observed in the secondary outcomes: health-related quality of life (−0.84, 95% CI: −5.33, 3.64, *p* = 0.713; gastrointestinal-specific anxiety (1.69, 95% CI: −0.89, 4.27, *p* = 0.201); last week’s worst pain intensity (−0.02, 95% CI: −1.13, 1.10, *p* = 0.977); anxiety symptoms (2.06, 95% CI: −0.22, 4.35, *p* = 0.079); and depressive symptoms (0.43, 95% CI: −0.45, 1.31, *p* = 0.339). Figure 2 illustrates rates of change for both groups from baseline to follow-up after iCBT.

The rates of change for the group without psychiatric comorbidity were nonsignificant from follow-up immediately after treatment to 6 months follow-up for the secondary outcomes: health-related quality of life (0.19, 95% CI: −2.30, 2.68, *p* = 0.881); gastrointestinal-specific anxiety (−0.059, 95% CI: −2.03, 0.85, *p* = 0.422); anxiety symptoms (0.30, 95% CI: −0.98, 1.57, *p* = 0.646); and depressive symptoms (0.03, 95% CI: −0.46, 0.52, *p* = 0.916). For last week’s worst pain intensity, there was a significant improvement from follow-up immediately after treatment to 6 months follow-up, −1.07 (95% CI: −1.69, −0.45, *p* < 0.001).

No significant differences between groups with and without psychiatric comorbidity in rates of change between follow-up immediately after treatment to 6 months follow-up were observed in any of the secondary outcomes: health-related quality of life (1.15, 95% CI: −3.44, 5.73, *p* = 0.625; gastrointestinal-specific anxiety (−0.34, 95% CI: −2.98, 2.30, *p* = 0.801); last week’s worst pain intensity (0.44, 95% CI: −0.70, 1.58, *p* = 0.450); anxiety symptoms (−0.78, 95% CI: −3.12, 1.56, *p* = 0.514); and depressive symptoms (−0.70, 95% CI: −1.60, 0.20, *p* = 0.130). Detailed descriptive information and model output for all secondary outcomes are presented in Supplemental Information 2.

## Discussion

In this exploratory study, we compared rates of change in treatment effects during and after iCBT for children with FAPDs, stratified by the presence or absence of psychiatric comorbidity. While children with psychiatric comorbidity exhibited more severe baseline symptoms across all measures, both groups demonstrated similar rates of improvement during treatment and from the end of treatment to follow-up.

These results contrast with findings observed in previous studies on chronic pain. In adult chronic pain patients, it has been shown that depression was associated with more pain complaints and a lower likelihood of recovery.^36^ They found that the presence of depression predicted not only musculoskeletal complaints but also future pain episodes, including low back pain, chest pain, and headaches. It has been speculated that the negative interaction between pain and, for example, depression, may be due to a shared underlying neurobiological mechanism, meaning that pain and negative affect are highly dependent.^36,37^ In line with these previous findings, a prospective cohort study following approximately 500,000 individuals showed mental illness and chronic pain to have a bidirectional influence with similar incidence rate ratios.

A possible explanation for the results in the present study could be that the core components of the treatment, including exposure, specifically target and improve abdominal symptoms—including pain regulation—by acting on the gut-brain axis, which may benefit both groups, regardless of psychiatric comorbidities. It is also possible that different factors are influencing the two groups, leading to similar outcomes as observed in this study. In the comorbid group, a higher overall symptom burden may provide greater potential for improvement. Conversely, the non-comorbid group, starting from a better position with a lower overall symptom burden, may have more favorable conditions for therapeutic change. Thus, the potential for improvement may differ between the two groups, yet result in similar treatment outcomes. Understanding these dynamics could have implications for tailoring interventions to maximize therapeutic benefits for these children.

Regarding the favorable changes in symptoms of anxiety and gastrointestinal anxiety, it could be that the exposure component in our iCBT may act on both. Given the well-documented efficacy of exposure therapy in the treatment of anxiety disorders,^38,39^ this would be plausible. The primary components of iCBT include exposure to gastrointestinal symptoms and situations that trigger gastrointestinal-specific anxiety. Research has demonstrated that gastrointestinal-specific anxiety has a unique explanatory role in the development and persistence of FAPDs, independent of other forms of anxiety.^40^ In our previous study of CBT in adult patients with fibromyalgia, we also found a clear reduction of anxiety in the group who received CBT.^41^ Our observations here indicate a reduction in both gastrointestinal-specific anxiety and broader measures of anxiety across both groups. This suggests that while the intervention specifically targets gastrointestinal-specific anxiety, the learning effects and coping skills acquired may generalize to other forms of anxiety.

A possible explanation for the observed improvement in depressive symptoms is that patients, through increased engagement in daily activities—such as physical exercise, attending school, and social interactions—gain access to natural reinforcers, thereby reducing depressive symptoms. The treatment also involves the child’s parents, supporting them in establishing a reinforcing context that motivates the child to participate in meaningful activities, such as school and extracurriculars, thereby increasing exposure to naturally reinforcing experiences. This process aligns with the principles of behavioral activation, which is known to be effective in treating adults with depression and shows promise for children.^42–44^

### Implications

This study provides the first evidence that exposure-based iCBT may effectively reduce abdominal symptoms in children with FAPDs, also in the presence of psychiatric comorbidity. Given that psychiatric conditions such as anxiety and depression are highly prevalent among children with FAPDs, these findings may offer valuable guidance for clinicians. Our study suggests that iCBT can be a viable and beneficial treatment option for this patient group, helping to alleviate both gastrointestinal and comorbid psychiatric disorders.

While children with psychiatric comorbidities experienced symptom improvement comparable to those without comorbidities, it is important to note that, given their higher symptom levels at baseline, post-treatment symptom levels remained higher compared to those without comorbidities. This indicates that while iCBT is effective, children with comorbid psychiatric conditions may still face a higher burden of abdominal symptoms as well as psychiatric symptoms. The observed transdiagnostic benefits of iCBT—improvements beyond gastrointestinal symptoms—may be clinically meaningful, as they contribute to reducing overall suffering. However, the lingering severity of symptoms underscores the need for additional or adjunctive interventions to further address their multifaceted challenges.

The need to address psychiatric comorbidity in chronic pain patients has previously been highlighted,^45^ and future studies should explore treatment approaches that target both gastrointestinal and psychiatric symptoms. Such approaches could include enhanced iCBT protocols, for example, in the form of added treatment modules targeting comorbid anxiety. By addressing both domains, further improvements could be made in these children’s well-being.

### Limitations

This secondary data analysis was conducted on data from both a feasibility study and an RCT. This was done from a pragmatic perspective, utilizing the comparable data our research team had collected in clinical studies up to this point. Given that the data in the analysis comes from a non-randomized sample, the integrity of our results is somewhat diminished, and confounding variables may have influenced the outcomes.

The sample size was a limitation in this study, compounded by the uneven group sizes, with only 36 individuals having psychiatric comorbidity. Given that the two groups received the same treatment, potential differences between the groups could be relatively small. Therefore, we cannot rule out that with a larger sample, small differences in effect may have been detected between the groups.

Also, due to limited statistical power, further exploratory subgroup analyses were not conducted. Consequently, we lack insights into potential differences in treatment effects across various psychiatric conditions. For the same reason, it was also not possible to address potential additive effects of multiple comorbid diagnoses.

This study focused specifically on children aged 8–12, which may limit the generalizability of the findings to adolescents aged 13–18, given the developmental differences between children and adolescents—such as cognitive maturity and emotional regulation. Additionally, the social contexts differ significantly across age groups. Adolescents face distinct stressors, such as academic pressures, social relationships, and emerging independence. Parents serve as an important contextual factor for treatment success in iCBT for both children and adolescents, but the parental role is particularly critical for young children. The importance of parental influence was highlighted by a mediation analysis, conducted by our research team, of an RCT with 90 parent-children dyads that underwent iCBT for FAPDs.^46^ It showed that parents’ levels of catastrophizing about their children’s symptoms had a mediating effect on their children’s treatment outcomes. It is possible that differences in parental influence and levels of parental involvement in treatment—between parents of children and parents of adolescents—may differentially affect youths’ ability to engage with and adhere to treatment, particularly when facing comorbid psychiatric challenges.

Taken together, these factors may impact the manifestation and management of FAPDs and psychiatric comorbidity differently across age groups. To address this limitation, future research should include both children and adolescents to examine whether the impact of psychiatric comorbidity on the efficacy of iCBT varies across these age groups.

Another limitation of this study is its reliance on self-reported online measures completed by children. Self-reporting, particularly in younger populations, can introduce several potential biases. Children aged 8–12 may have limited insight into their symptoms or difficulty accurately depicting their experiences, which could lead to response biases, recall inaccuracies, or social desirability effects. However, given FAPD’s lack of reliable biologic markers, patient-reported outcome measures of symptoms are the recommended measurement instruments to investigate the effect of treatment in clinical trials.^47^ In future studies, this could be addressed by adding more objective measures of gastrointestinal symptoms, such as clinician ratings or quantitative sensory testing methods, to examine pain perception.

One additional limitation of the study is that the psychiatric assessment was conducted at the time of inclusion in the study. Consequently, for approximately one-third of the sample (the iCBT-delayed group), the psychiatric diagnostic screening occurred nearly 3 months prior to the baseline assessment. Hence, it cannot be ruled out that some children’s diagnostic status might have changed at the time of the study’s baseline measurements.

Taken together, these findings should be interpreted with caution. To further investigate the impact of psychiatric comorbidity on psychological treatment outcomes for patients with FAPDs, future research should employ an RCT design with sufficient power to detect differences between children with psychiatric comorbidity across different study arms. In addition, such a study could grade psychiatric complexity based on factors such as symptom severity or the number of diagnoses.

## Conclusion

The results of this study demonstrate that iCBT seems to be beneficial for children with FAPDs, even in the presence of mild to moderate psychiatric comorbidity. This is an encouraging result, given the high prevalence of concurrent anxiety and depression in children with FAPDs, and will assist clinicians in prioritizing treatment strategies for managing functional abdominal pain.

## Supplemental Material

sj-docx-4-tag-10.1177_17562848251384605 – Supplemental material for Comparing the treatment effects of online cognitive-behavioral therapy for pediatric functional abdominal pain disorders with and without psychiatric comorbidity Supplemental material, sj-docx-4-tag-10.1177_17562848251384605 for Comparing the treatment effects of online cognitive-behavioral therapy for pediatric functional abdominal pain disorders with and without psychiatric comorbidity by Viktor Vadenmark Lundqvist, Aleksandra Bujacz, Jenny Rickardsson, Johan Åhlén, Martin Jonsjö, Jörgen Rosén, Sarah Vigerland, Karin Jensen, Marianne Bonnert and Maria Lalouni in Therapeutic Advances in Gastroenterology

sj-pdf-1-tag-10.1177_17562848251384605 – Supplemental material for Comparing the treatment effects of online cognitive-behavioral therapy for pediatric functional abdominal pain disorders with and without psychiatric comorbidity Supplemental material, sj-pdf-1-tag-10.1177_17562848251384605 for Comparing the treatment effects of online cognitive-behavioral therapy for pediatric functional abdominal pain disorders with and without psychiatric comorbidity by Viktor Vadenmark Lundqvist, Aleksandra Bujacz, Jenny Rickardsson, Johan Åhlén, Martin Jonsjö, Jörgen Rosén, Sarah Vigerland, Karin Jensen, Marianne Bonnert and Maria Lalouni in Therapeutic Advances in Gastroenterology

sj-pdf-2-tag-10.1177_17562848251384605 – Supplemental material for Comparing the treatment effects of online cognitive-behavioral therapy for pediatric functional abdominal pain disorders with and without psychiatric comorbidity Supplemental material, sj-pdf-2-tag-10.1177_17562848251384605 for Comparing the treatment effects of online cognitive-behavioral therapy for pediatric functional abdominal pain disorders with and without psychiatric comorbidity by Viktor Vadenmark Lundqvist, Aleksandra Bujacz, Jenny Rickardsson, Johan Åhlén, Martin Jonsjö, Jörgen Rosén, Sarah Vigerland, Karin Jensen, Marianne Bonnert and Maria Lalouni in Therapeutic Advances in Gastroenterology

sj-pdf-3-tag-10.1177_17562848251384605 – Supplemental material for Comparing the treatment effects of online cognitive-behavioral therapy for pediatric functional abdominal pain disorders with and without psychiatric comorbidity Supplemental material, sj-pdf-3-tag-10.1177_17562848251384605 for Comparing the treatment effects of online cognitive-behavioral therapy for pediatric functional abdominal pain disorders with and without psychiatric comorbidity by Viktor Vadenmark Lundqvist, Aleksandra Bujacz, Jenny Rickardsson, Johan Åhlén, Martin Jonsjö, Jörgen Rosén, Sarah Vigerland, Karin Jensen, Marianne Bonnert and Maria Lalouni in Therapeutic Advances in Gastroenterology

## Declarations

**Ethics approval and consent to participate:** The study was approved by the Swedish Ethical Review Authority: 2016/1289-31 and 2023-03783-02. OSF Registration: https://doi.org/10.17605/OSF.IO/QTR65. Informed consent to participate was obtained orally by the children and in written form by all caregivers in the original studies.

**Consent for publication:** Not applicable for this study.

**Availability of data and materials:** The data presented in this article are not readily available because we do not have an ethical permit to share the data. Requests to access the data should be made to maria.lalouni@ki.se.
