# Supplementary material for: Comparing the treatment effects of online cognitive-behavioral therapy for pediatric functional abdominal pain disorders with and without psychiatric comorbidity
Source: Ther Adv Gastroenterol. 2025 Oct 9;18:17562848251384605. doi: 10.1177/17562848251384605 (PMC12515282; doi:10.1177/17562848251384605)
Supplement: sj-docx-4-tag-10.1177_17562848251384605 – Supplemental material for Comparing the treatment effects of online cognitive-behavioral therapy for pediatric functional abdominal pain disorders with and without psychiatric comorbidity [file sj-docx-4-tag-10.1177_17562848251384605.docx]

Supplementary table. Observed means and standard deviations for the comorbid group (n=36) and the non-comorbid group (n=84) at baseline, follow-up after treatment, and six-month follow-up.

|  | **Baseline** | **Follow-up**  **after iCBT** | **Six-month**  **follow-up** |
| --- | --- | --- | --- |
| **PedsQL Gastro, M (SD)** |  |  |  |
| Comorbid | 57.7 (15.3) | 67.9 (16.9) | 71.4 (15.0) |
| Non-comorbid | 63.2 (13.0) | 77.5 (14.6) | 77.7 (16.9) |
| **Peds QL QoL, M (SD)** |  |  |  |
| Comorbid | 69.5 (14.9) | 79.1 (11.1) | 81.2 (11.3) |
| Non-comorbid | 78.2 (11.6) | 87.6 (10.8) | 87.9 (10.6) |
| **VSI, M (SD)** |  |  |  |
| Comorbid | 14.0 (9.2) | 8.9 (6.8) | 7.8 (6.5) |
| Non-comorbid | 10.7 (7.0) | 4.4 (4.1) | 3.7 (3.7) |
| **FACES, M (SD)** |  |  |  |
| Comorbid | 6.8 (1.9) | 5.5 (2.1) | 4.8 (2.4) |
| Non-comorbid | 5.8 (2.4) | 4.5 (2.9) | 3.4 (2.7) |
| **SCAS, M (SD)** |  |  |  |
| Comorbid | 17.8 (9.7) | 15.5 (7.6) | 15.3 (8.6) |
| Non-comorbid | 11.3 (7.4) | 8.2 (5.9) | 8.7 (6.6) |
| **CDI, mean (SD)** |  |  |  |
| Comorbid | 4.3 (3.6) | 3.7 (3.1) | 2.9 (3.0) |
| Non-comorbid | 2.3 (2.2) | 1.4 (2.0) | 1.5 (2.2) |

*Abbreviations*: PedsQL Gastro, Pediatric Quality of Life Gastrointestinal Symptom Scale (PedsQL Gastro); Peds QL QoL, Pediatric Quality of Life Inventory; VSI, Visceral Sensitivity Index; FACES, Faces Pain Rating Scale; SCAS-S, Spence Children Anxiety Scale - Short version; CDI-S, Child Depression Inventory - Short version.
