# Supplementary material for: Comparing the treatment effects of online cognitive-behavioral therapy for pediatric functional abdominal pain disorders with and without psychiatric comorbidity
Source: Ther Adv Gastroenterol. 2025 Oct 9;18:17562848251384605. doi: 10.1177/17562848251384605 (PMC12515282; doi:10.1177/17562848251384605)
Supplement: sj-pdf-1-tag-10.1177_17562848251384605 – Supplemental material for Comparing the treatment effects of online cognitive-behavioral therapy for pediatric functional abdominal pain disorders with and without psychiatric comorbidity [file sj-pdf-1-tag-10.1177_17562848251384605.pdf]

# Results

N = 120
One child was excluded after the child's parent reported that the child had answered the assessments without reading the questions.
The study uses secondary data from a feasibility trial and a randomized controlled trial (RCT) in which children aged 8-12 years with functional abdominal pain disorders (FAPDs) received internet-delivered cognitive behavioral therapy (ICBT) for their gastrointestinal problems.

# Descriptives

Descriptives

| | STUDY_GROUP | COMORB |
|---|---|---|
| N | 120 | 120 |
| Missing | 1 | 1 |

## Frequencies

Frequencies of STUDY_GROUP

| Levels | Counts | % of Total | Cumulative % |
|---|---|---|---|
| 1 | 31 | 25.8 % | 25.8 % |
| 2 | 45 | 37.5 % | 63.3 % |
| 3 | 44 | 36.7 % | 100.0 % |

1=feasibility study
2=RCT Internet CBT group
3= RCT Treatment as usual group after being crossed over to Internet-CBT
All children received ICBT in one of the following ways:
1. Feasibility study[2]. All children received ICBT after inclusion.
2. RCT, ICBT group[3]. All children randomized to the ICBT group received ICBT directly after the randomization.
3. RCT, treatment as usual group[3]. The children randomized to treatment as usual was crossed over to ICBT after 10 weeks of treatment as usual. Their assessments when taking part of ICBT will be used in the study. However, the psychiatric comorbidity was assessed before randomization.

Frequencies of COMORB

| Levels | Counts | % of Total | Cumulative % |
|---|---|---|---|
| 0 | 84 | 70.0 % | 70.0 % |
| 1 | 36 | 30.0 % | 100.0 % |

0=no comorbidity 1=psychiatric comorbidity
All children will be divided into two groups: presence or absence of psychiatric comorbidity, based on the psychologist's psychiatric assessment and additional parental reports of psychiatric and/or neuropsychiatric diagnosis at baseline. The outcomes of the two groups will be compared.
Assessment of the independent variable: psychiatric comorbidity
Mini International Neuropsychiatric Interview for Children and Adolescents (MINI-KID)[5] was assessed by a psychologist at baseline in a structured interview with the child.
The MINI- KID was assessed at the clinic with the aim to determine psychiatric comorbidity. All interviewers were clinical psychologists. Parents were also asked at baseline whether their children had any current psychiatric or neuropsychiatric disorder, diagnosed elsewhere. The two groups for which the outcome variables will be compared will be categorized as either presence or absence of a psychiatric or a neuropsychiatric disorder (by MINI-KID and/or parent report).

# Descriptives

PRIMARY OUTCOME

Descriptives

| | QLGASTRO_PRE | QLGASTRO_W1 | QLGASTRO_W2 | QLGASTRO_W3 | QLGASTRO_W4 | QLGASTRO_W5 | QLGASTRO_W6 | QLGASTRO_W7 | QLGASTRO_W8 | QLGAST |
|---|---|---|---|---|---|---|---|---|---|---|
| N | 118 | 115 | 76 | 115 | 73 | 108 | 72 | 105 | 63 | |
| Missing | 3 | 6 | 45 | 6 | 48 | 13 | 49 | 16 | 58 | |
| Mean | 61.7 | 67.7 | 68.5 | 68.5 | 71.3 | 71.1 | 74.5 | 71.7 | 75.0 | |
| Standard deviation | 13.8 | 14.2 | 12.5 | 14.4 | 15.3 | 16.1 | 14.9 | 16.4 | 16.1 | |
| Minimum | 25.0 | 33.3 | 36.1 | 27.8 | 30.6 | 25.0 | 30.6 | 33.3 | 33.3 | |
| Maximum | 94.4 | 100 | 97.2 | 100 | 94.4 | 100 | 100 | 100 | 100 | |
| Skewness | -0.350 | -0.150 | -0.114 | -0.181 | -0.414 | -0.252 | -0.643 | -0.549 | -0.837 | |
| Std. error skewness | 0.223 | 0.226 | 0.276 | 0.226 | 0.281 | 0.233 | 0.283 | 0.236 | 0.302 | |

The primary dependent variable was self-assessed gastrointestinal symptoms using the Pediatric Quality of Life Gastrointestinal Symptom Scale (PedsQL Gastro)[6]. The PedsQL Gastro was assessed by the children via an internet platform at baseline, at follow-up after ICBT, and at follow-up six months after treatment completion. These assessments used a one month recall period. The PedsQL Gastro was also assessed weekly during the 10 week's treatment using a one week's recall period. In the RCT, the children randomized to treatment as usual assessed PedsQL Gastro bi-weekly after they crossed over to ICBT (the time period of interest in this study for this group). Allowed range 0-100.

# Descriptives

secondary outcome: Depressive symptoms were self-assessed by the child using the Child Depression Inventory - Short version (CDI-S). Depression scale at baseline/post/6m follow up, allowed range 0-20

Descriptives

| | CDI_PRE | CDI_POST | CDI_FU6 |
|---|---|---|---|
| N | 118 | 111 | 109 |
| Missing | 3 | 10 | 12 |
| Mean | 2.86 | 2.10 | 1.89 |
| Standard deviation | 2.82 | 2.60 | 2.53 |
| Minimum | 0 | 0 | 0 |
| Maximum | 14 | 12 | 11 |
| Skewness | 1.35 | 1.59 | 1.57 |
| Std. error skewness | 0.223 | 0.229 | 0.231 |

## Descriptives

secondary outcome: Gastrointestinal-specific anxiety was self-assessed by the child using the Visceral Sensitivity Index – Child-adapted version, allowed range 0-35

Descriptives

| | VSI_PRE | VSI_POST | VSI_FU6 |
|---|---|---|---|
| N | 118 | 111 | 109 |
| Missing | 3 | 10 | 12 |
| Mean | 11.6 | 5.74 | 4.90 |
| Standard deviation | 7.78 | 5.46 | 5.03 |
| Minimum | 0 | 0 | 0 |
| Maximum | 34 | 25 | 22 |
| Skewness | 0.686 | 1.17 | 1.27 |
| Std. error skewness | 0.223 | 0.229 | 0.231 |

## Descriptives

secondary outcome: Health-related quality of life was self-assessed by the child using the Pediatric Quality of Life Inventory (PedsQL QOL). allowed range 0-100

Descriptives

| | QLQOL_PRE | QLQOL_POST | QLQOL_FU6 |
|---|---|---|---|
| N | 118 | 111 | 110 |
| Missing | 3 | 10 | 11 |
| Mean | 75.8 | 85.1 | 86.0 |
| Standard deviation | 12.9 | 11.5 | 11.2 |
| Minimum | 39.1 | 55.2 | 48.8 |
| Maximum | 100 | 100 | 100 |
| Skewness | -0.664 | -0.674 | -0.716 |
| Std. error skewness | 0.223 | 0.229 | 0.230 |

## Descriptives

secondary outcome: Last week's worst pain intensity was self-assessed by the child using the Faces Pain Rating Scale (FACES). allowed range 0-10

Descriptives

| | FACES_PRE | FACES_POST | FACES_FU6 |
|---|---|---|---|
| N | 118 | 111 | 110 |
| Missing | 3 | 10 | 11 |
| Mean | 6.08 | 4.77 | 3.82 |
| Standard deviation | 2.28 | 2.73 | 2.68 |
| Minimum | 0 | 0 | 0 |
| Maximum | 10 | 10 | 10 |
| Skewness | -0.405 | -0.101 | 0.201 |
| Std. error skewness | 0.223 | 0.229 | 0.230 |

## Descriptives

secondary outcome: Anxiety symptoms were self-assessed by the child using the Spence Children Anxiety Scale - Short version (SCAS-S). allowed range 0-54

Descriptives

| | SCAS_PRE | SCAS_POST | SCAS_FU6 |
|---|---|---|---|
| N | 118 | 111 | 109 |
| Missing | 3 | 10 | 12 |
| Mean | 13.0 | 10.4 | 10.6 |
| Standard deviation | 7.88 | 7.22 | 7.82 |
| Minimum | 0 | 0 | 0 |
| Maximum | 41 | 30 | 29 |
| Skewness | 0.711 | 0.751 | 0.701 |
| Std. error skewness | 0.223 | 0.229 | 0.231 |

## References


**[1]** The jamovi project (2021). *jamovi*. (Version 2.2) [Computer Software]. Retrieved from https://www.jamovi.org.

**[2]** R Core Team (2021). *R: A Language and environment for statistical computing*. (Version 4.0) [Computer software]. Retrieved from https://cran.r-project.org. (R packages retrieved from MRAN snapshot 2021-04-01).
