# Supplementary material for: Comparing the treatment effects of online cognitive-behavioral therapy for pediatric functional abdominal pain disorders with and without psychiatric comorbidity
Source: Ther Adv Gastroenterol. 2025 Oct 9;18:17562848251384605. doi: 10.1177/17562848251384605 (PMC12515282; doi:10.1177/17562848251384605)
Supplement: sj-pdf-2-tag-10.1177_17562848251384605 – Supplemental material for Comparing the treatment effects of online cognitive-behavioral therapy for pediatric functional abdominal pain disorders with and without psychiatric comorbidity [file sj-pdf-2-tag-10.1177_17562848251384605.pdf]

Results

Descriptives

Descriptives

	comorb	N	Missing	Mean	Median	SD	Minimum	Maximum
gastro	0	876	216	73.4	72.2	15.1	16.7	100
	1	355	113	65.8	66.7	15.6	25.0	100

Flexplot

Analysis Plot

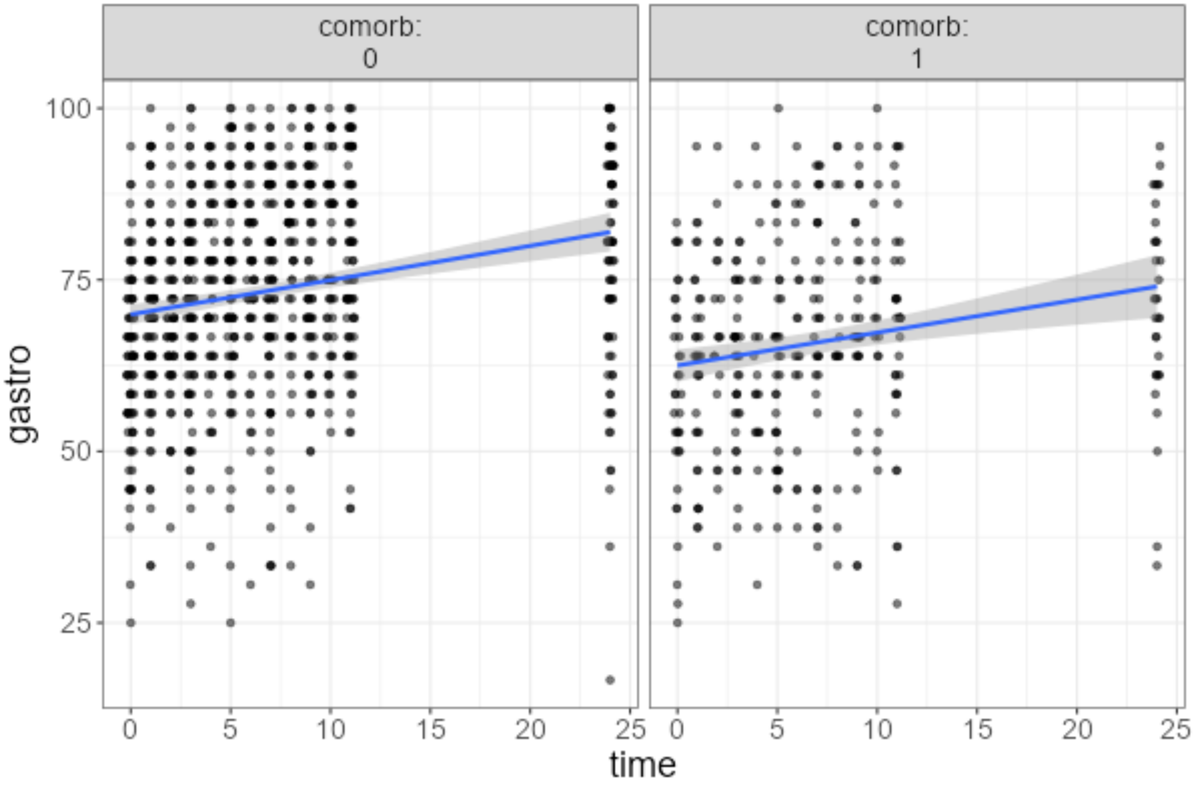

Note: Random effects are plotted by id

Mixed Model

Model Info

Info	
Estimate	Linear mixed model fit by REML
Call	gastro ~ 1 + ttreat + comorb + tfollow + ttreat:comorb + comorb:tfollow+(1 + ttreat | id)
AIC	8998.067
BIC	9043.148
LogLikel.	-4485.996
R-squared Marginal	0.104
R-squared Conditional	0.772
Converged	yes
Optimizer	bobyqa

[3]

Model Results

Fixed Effect Omnibus tests

	F	Num df	Den df	p
ttreat	79.496	1	134	< .001
comorb	9.985	1	123	0.002
tfollow	0.387	1	1015	0.534
ttreat ✻ comorb	2.005	1	134	0.159
comorb ✻ tfollow	1.197	1	1015	0.274

Note. Satterthwaite method for degrees of freedom

Fixed Effects Parameter Estimates

				95% Confidence Interval				
Names	Effect	Estimate	SE	Lower	Upper	df	t	p
(Intercept)	(Intercept)	78.979	1.668	75.711	82.247	122	47.36	< .001
ttreat	ttreat	1.073	0.112	0.853	1.293	130	9.56	< .001
comorb1	1 - 0	-9.710	3.073	-15.733	-3.687	123	-3.16	0.002
tfollow	tfollow	-1.627	1.022	-3.630	0.376	1013	-1.59	0.112
ttreat ✻ comorb1	ttreat ✻ 1 - 0	-0.294	0.208	-0.701	0.113	134	-1.42	0.159
comorb1 ✻ tfollow	1 - 0 ✻ tfollow	2.074	1.896	-1.642	5.791	1015	1.09	0.274

Random Components

Groups	Name	SD	Variance	ICC
id	(Intercept)	14.406	207.540	0.782
	ttreat	0.739	0.546	
Residual		7.615	57.995	

Note. Number of Obs: 1231 , groups: id 119

Random Parameters correlations

Groups	Param.1	Param.2	Corr.
id	(Intercept)	ttreat	0.541

Random Effect LRT

Test	N. par	AIC	LRT	df	p
ttreat in (1 + ttreat | id)	8	9040	52.5	2.00	< .001

Effects Plots

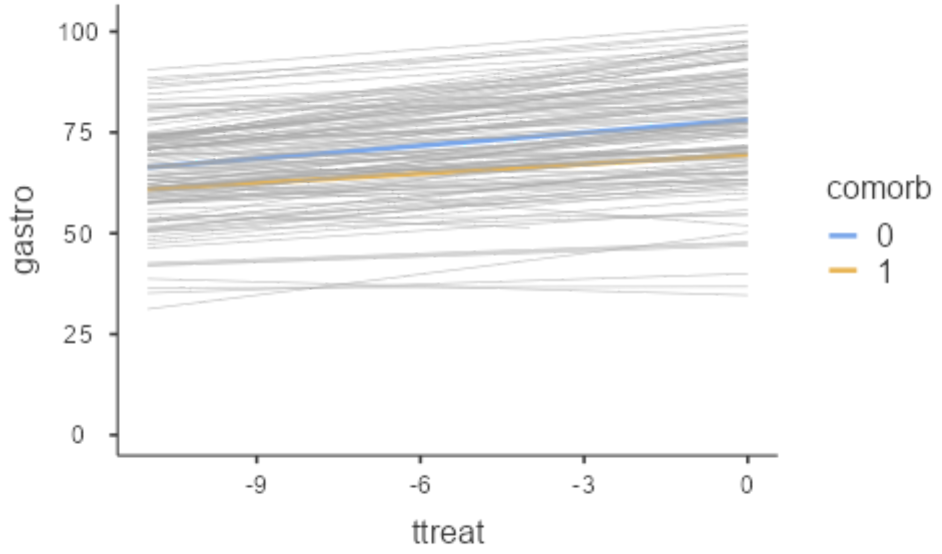

Note: Random effects are plotted by id

References

[1] The jamovi project (2021). *jamovi*. (Version 2.2) [Computer Software]. Retrieved from https://www.jamovi.org.

[2] R Core Team (2021). *R: A Language and environment for statistical computing*. (Version 4.0) [Computer software]. Retrieved from https://cran.r-project.org. (R packages retrieved from MRAN snapshot 2021-04-01).

[3] Gallucci, M. (2019). *GAMLj: General analyses for linear models*. [jamovi module]. Retrieved from https://gamlj.github.io/.
