# Supplementary material for: Comparing the treatment effects of online cognitive-behavioral therapy for pediatric functional abdominal pain disorders with and without psychiatric comorbidity
Source: Ther Adv Gastroenterol. 2025 Oct 9;18:17562848251384605. doi: 10.1177/17562848251384605 (PMC12515282; doi:10.1177/17562848251384605)
Supplement: sj-pdf-3-tag-10.1177_17562848251384605 – Supplemental material for Comparing the treatment effects of online cognitive-behavioral therapy for pediatric functional abdominal pain disorders with and without psychiatric comorbidity [file sj-pdf-3-tag-10.1177_17562848251384605.pdf]

Results

Descriptives

Descriptives

	comorb	dep	gastro	qol	faces	scas	vsi
N	0	238	876	239	239	238	238
	1	100	355	100	100	100	100
Missing	0	854	216	853	853	854	854
	1	368	113	368	368	368	368
Mean	0	1.75	73.4	84.5	4.59	9.46	6.36
	1	3.61	65.8	76.7	5.71	15.9	10.2
Median	0	1.00	72.2	86.9	5	8.00	5.00
	1	3.00	66.7	78.3	6.00	16.0	9.00
Standard deviation	0	2.16	15.1	11.8	2.82	6.81	6.10
	1	3.29	15.6	13.2	2.29	7.91	7.95
Minimum	0	0	16.7	44.7	0	0	0
	1	0	25.0	39.1	1	1	0
Maximum	0	9	100	100	10	41	28
	1	14	100	97.8	10	34	34

Flexplot

Analysis Plot

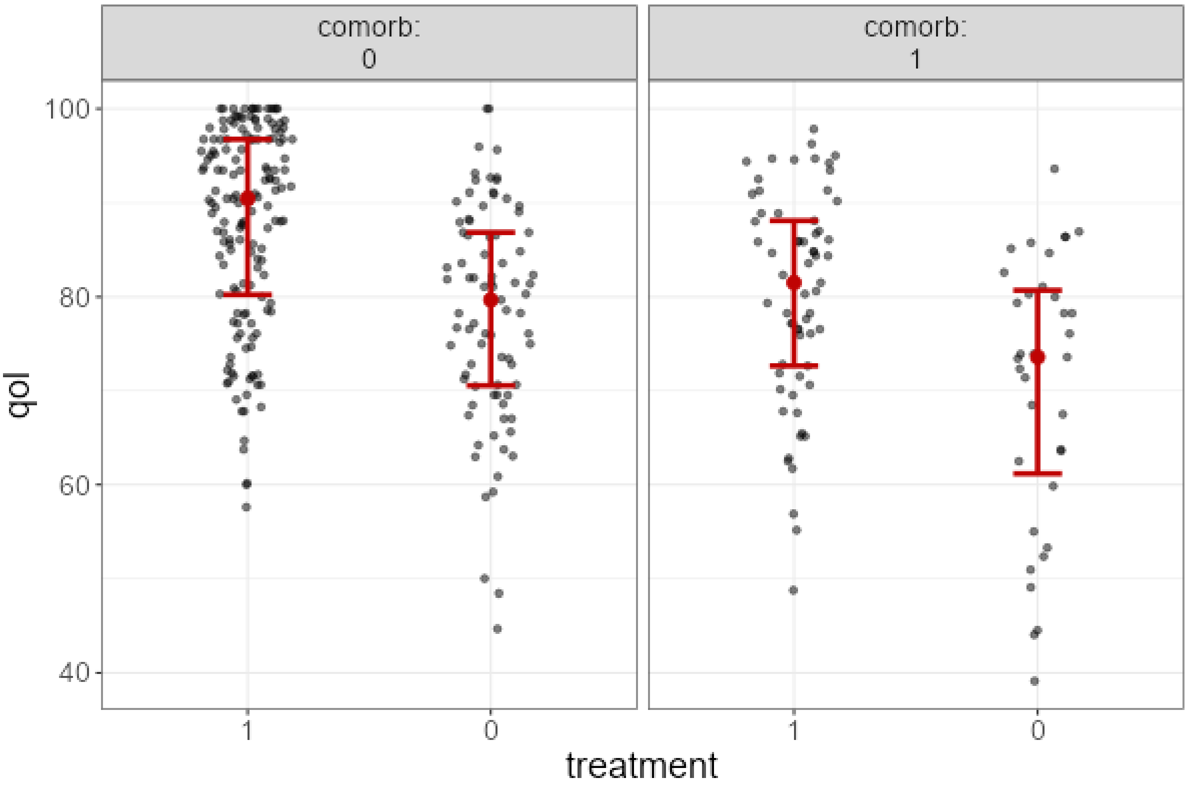

Flexplot

Analysis Plot

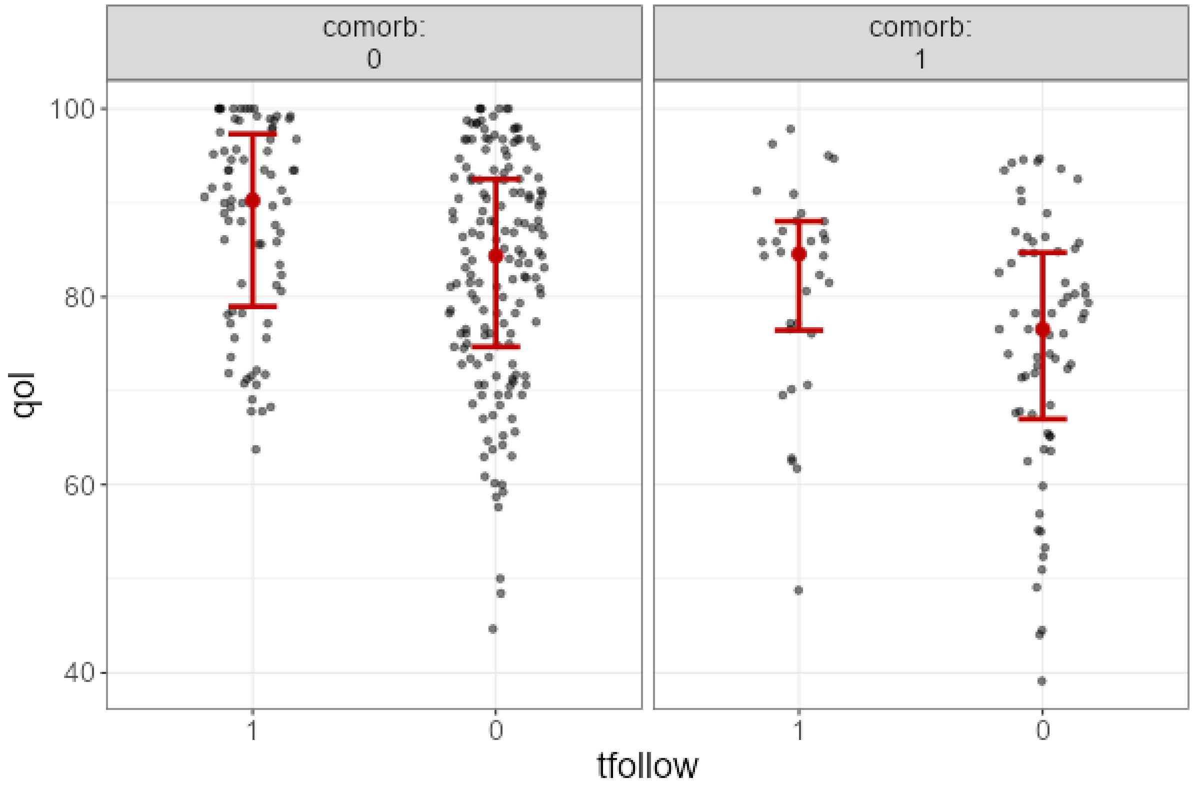

Flexplot

Analysis Plot

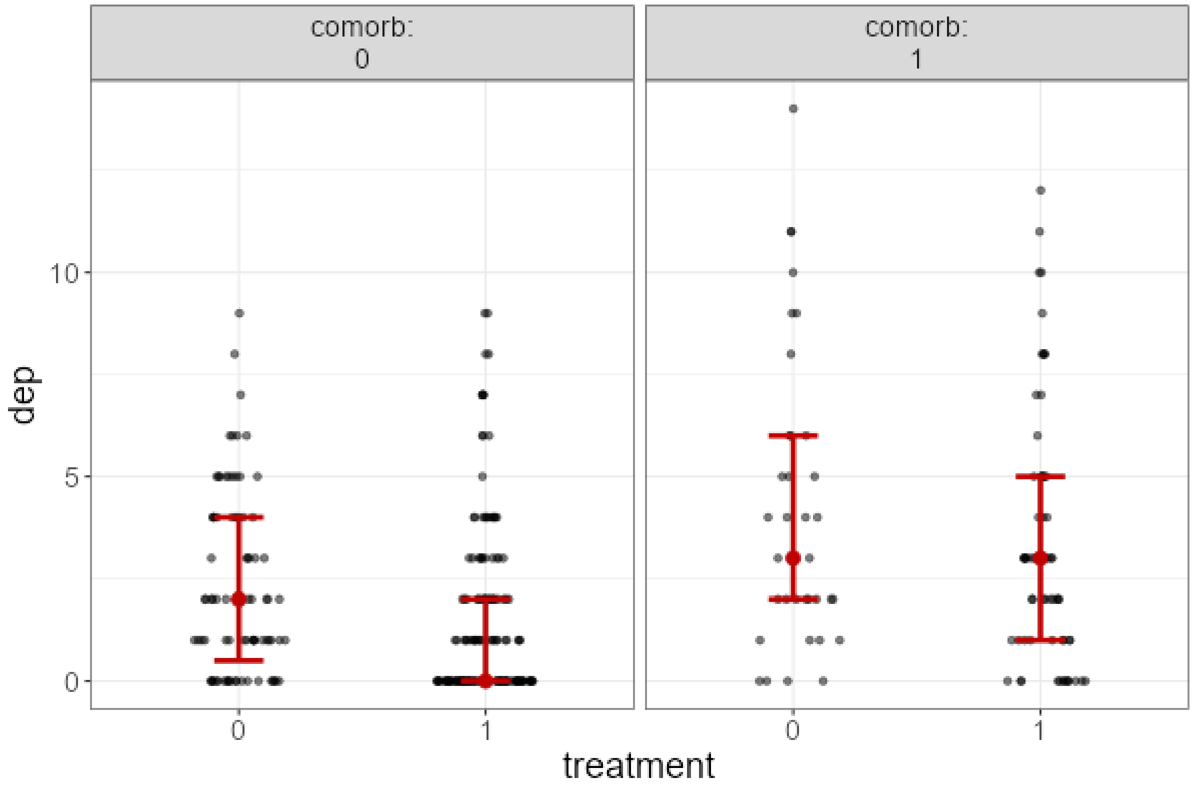

Flexplot

Analysis Plot

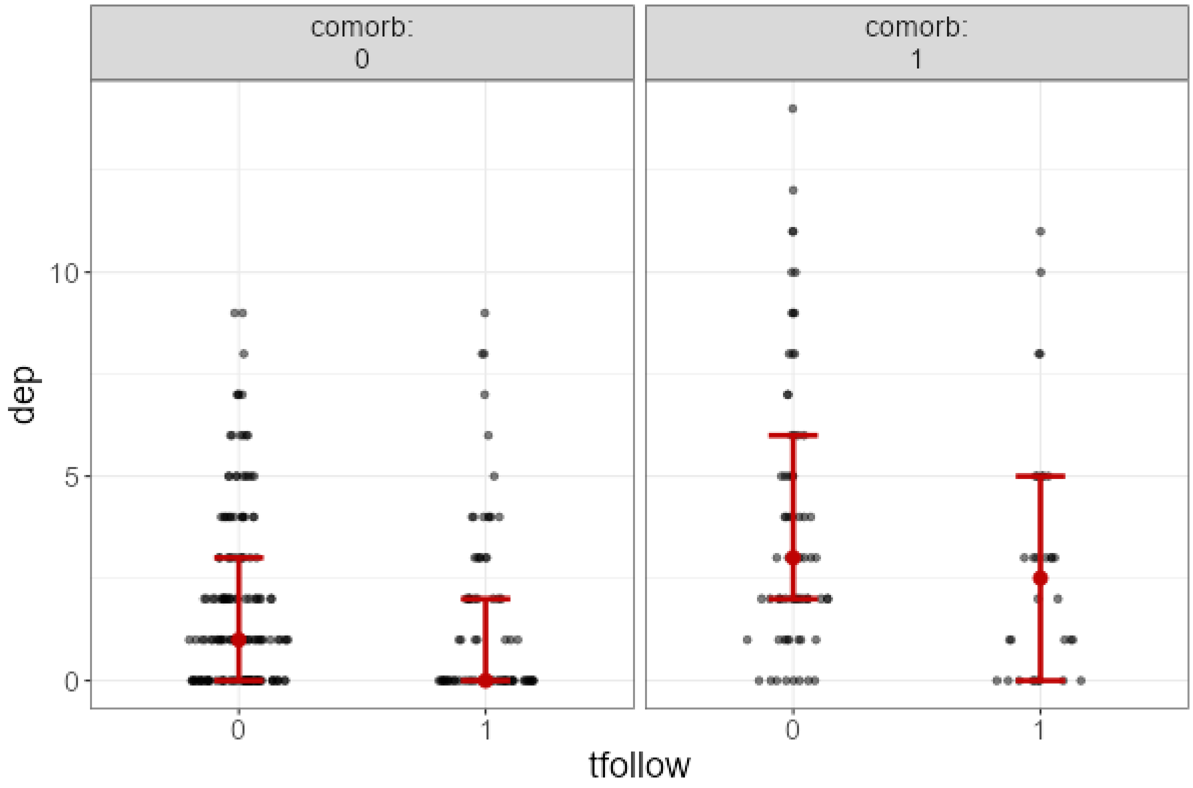

Flexplot

Analysis Plot

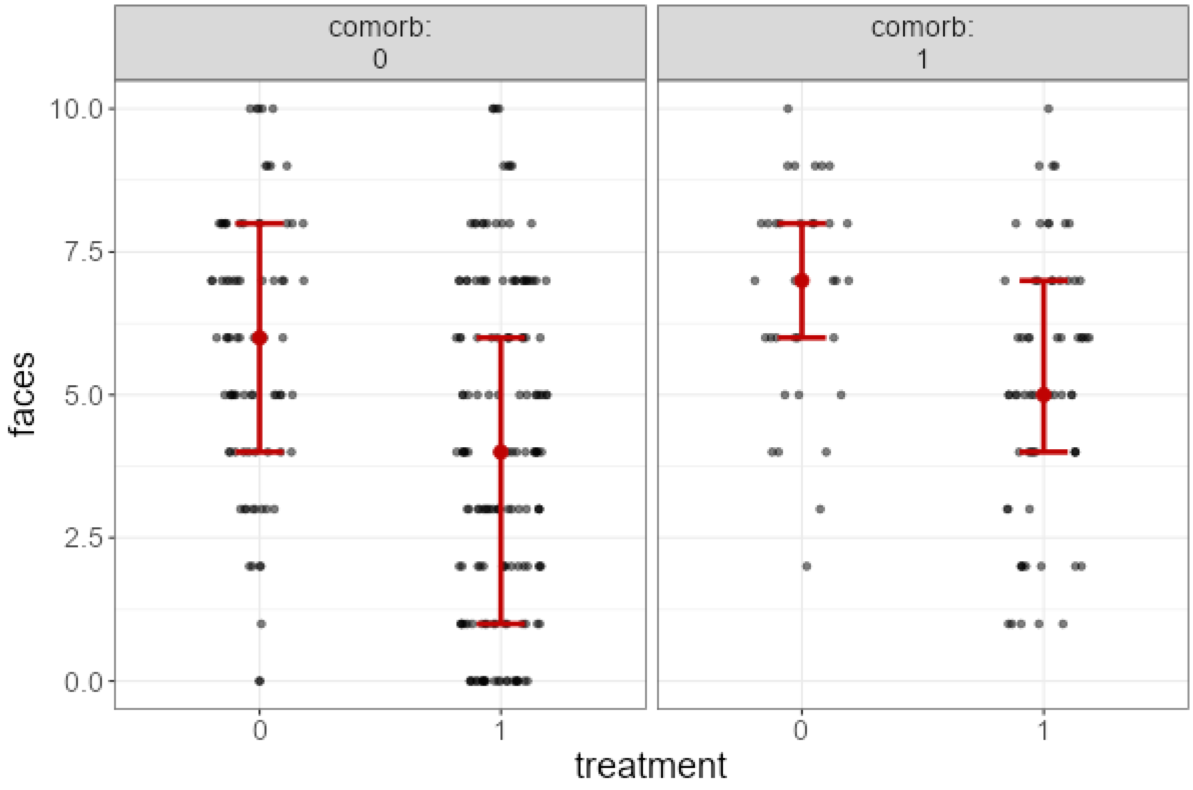

Flexplot

Analysis Plot

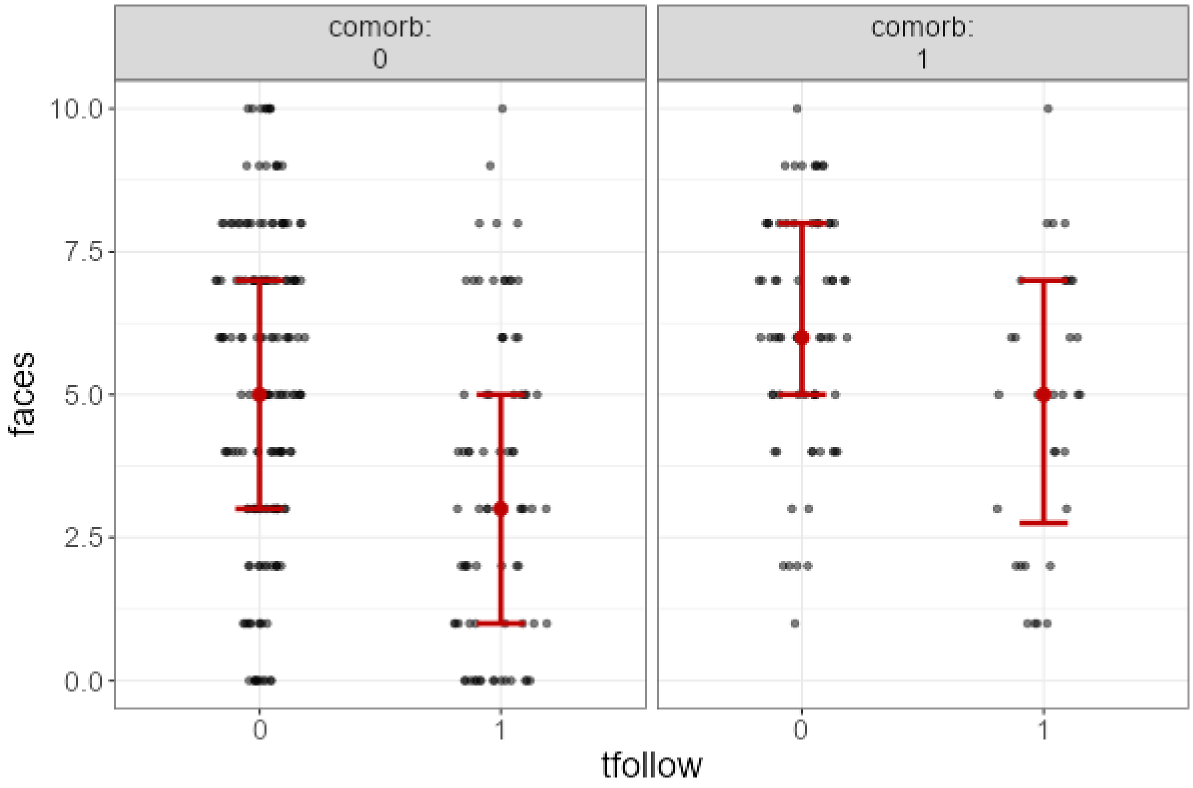

Flexplot

Analysis Plot

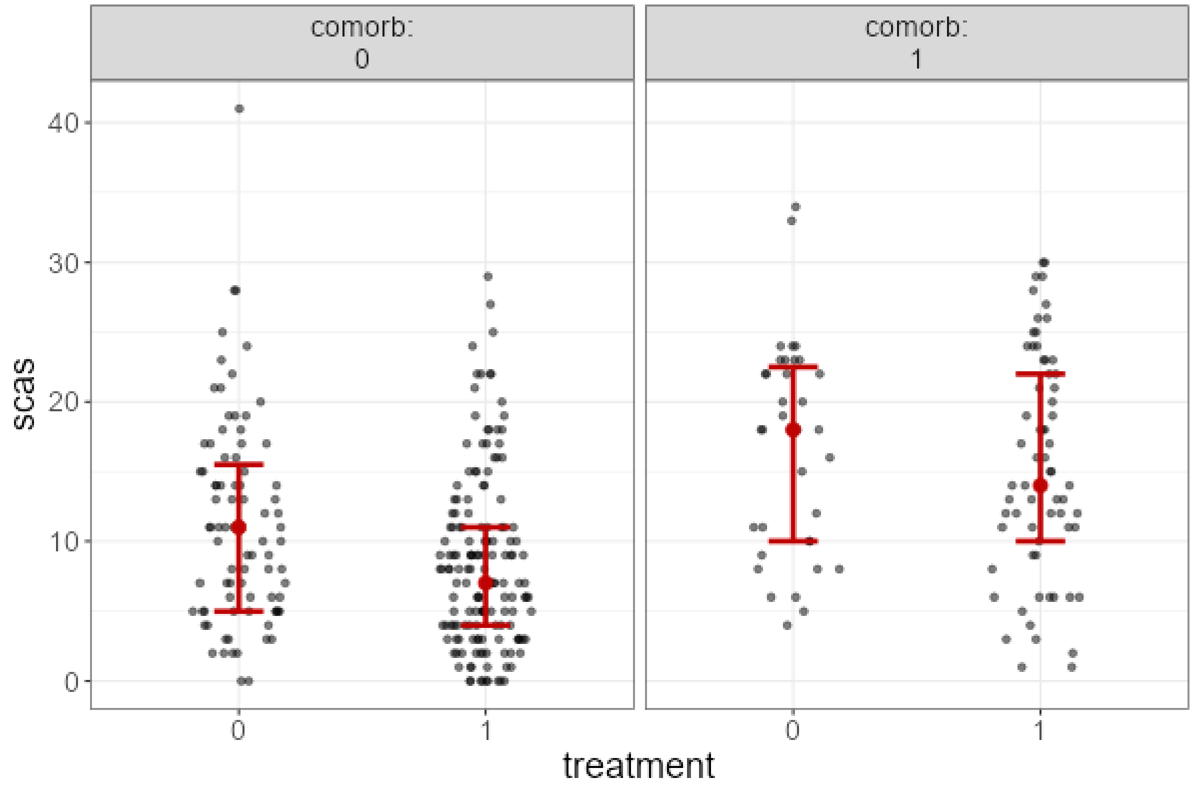

Flexplot

Analysis Plot

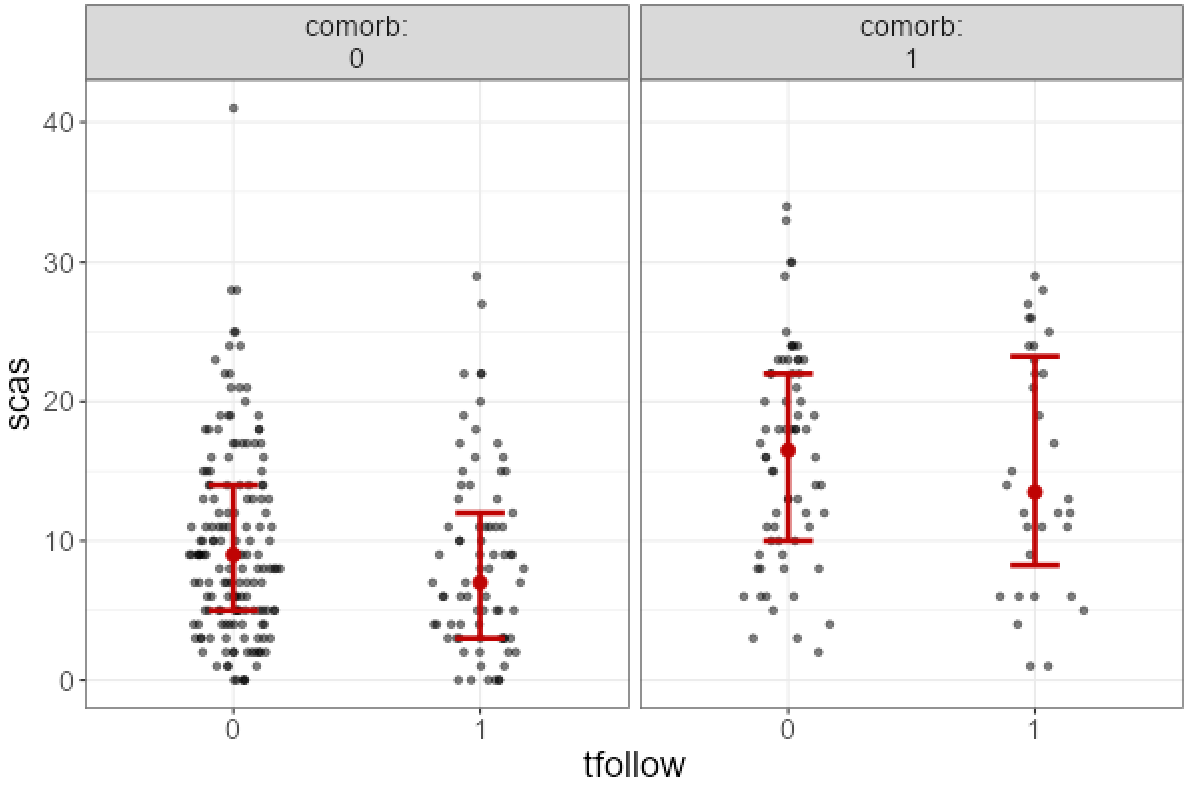

Flexplot

Analysis Plot

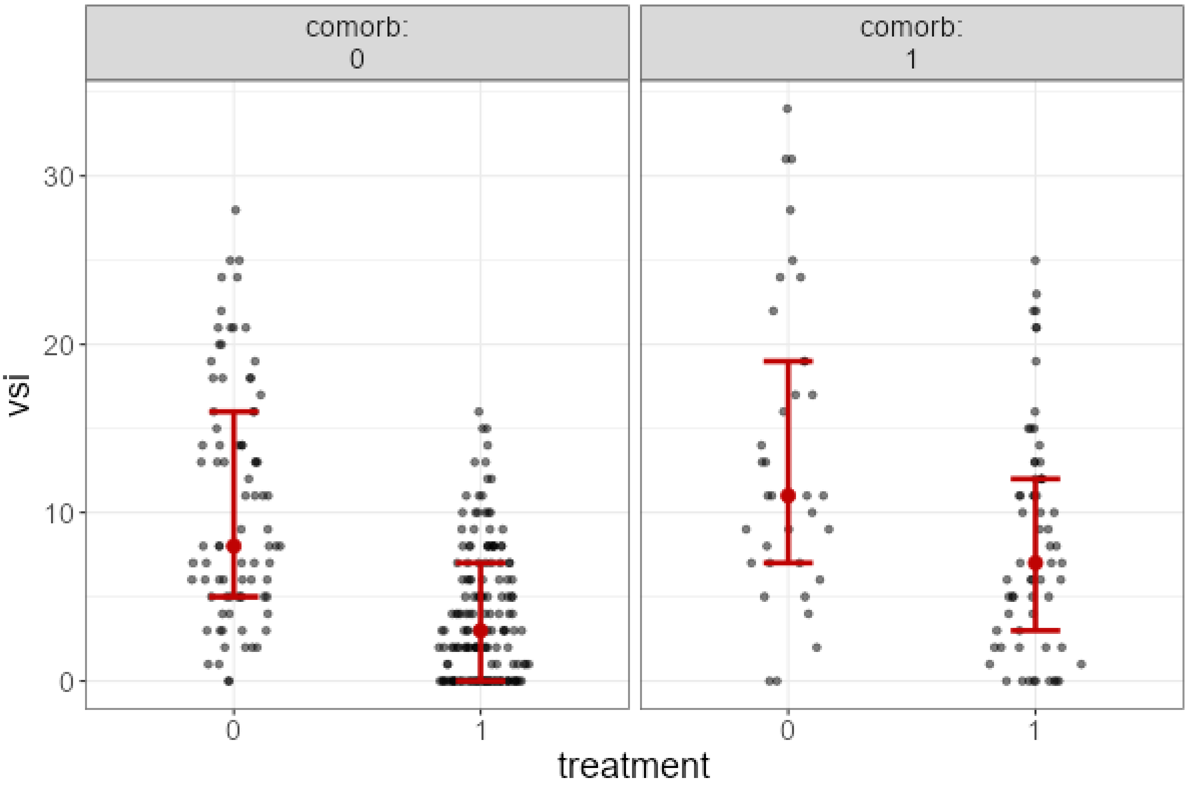

Flexplot

Analysis Plot

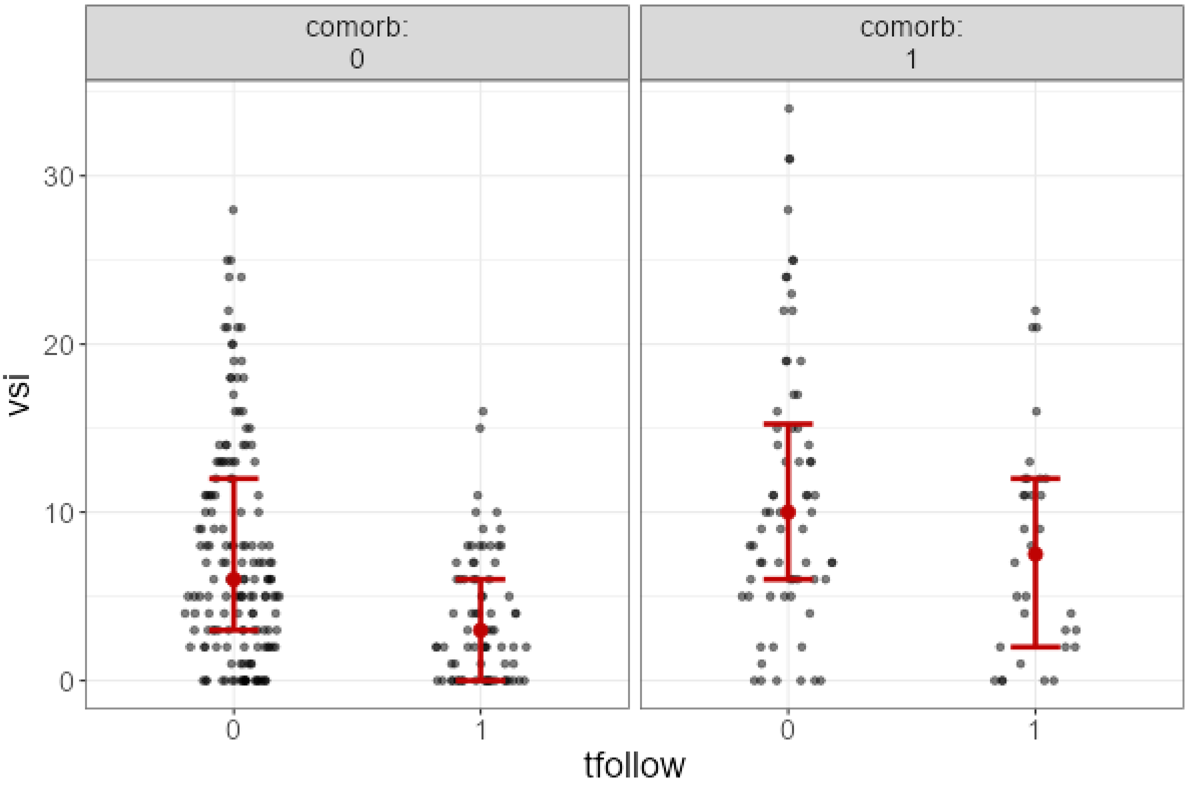

Flexplot

Analysis Plot

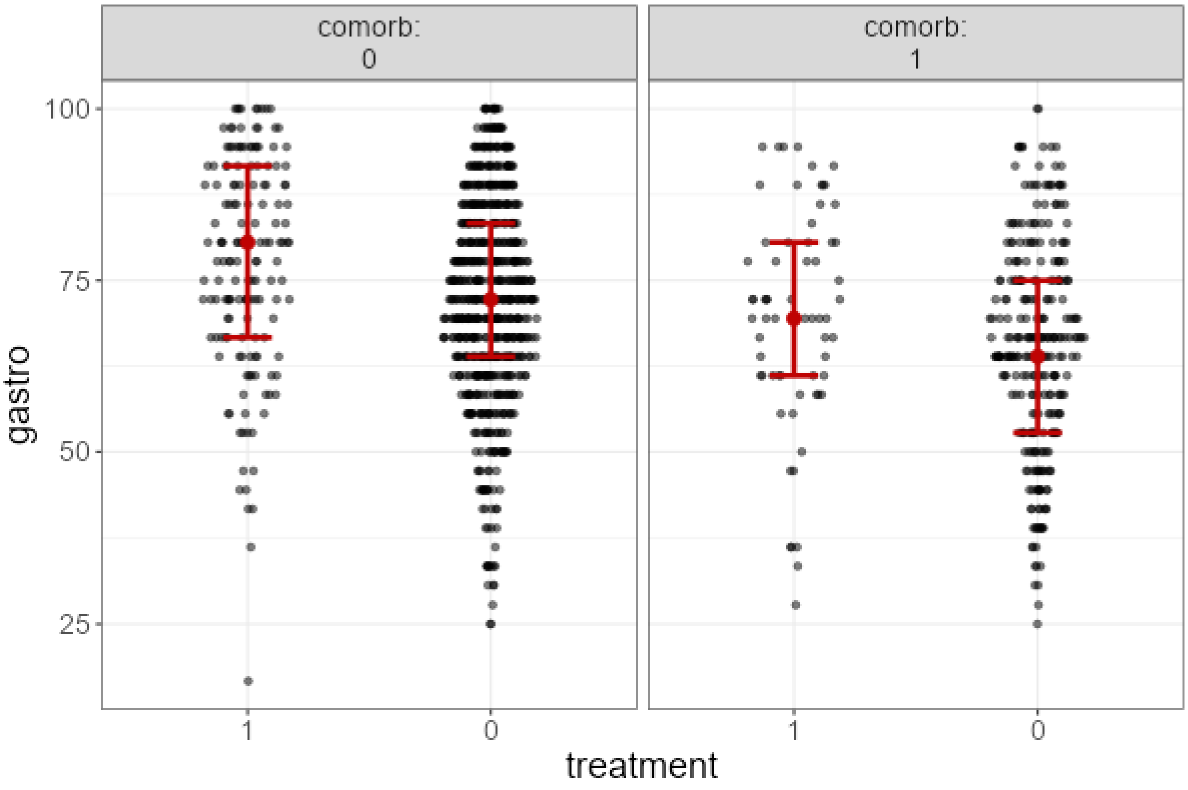

Flexplot

Analysis Plot

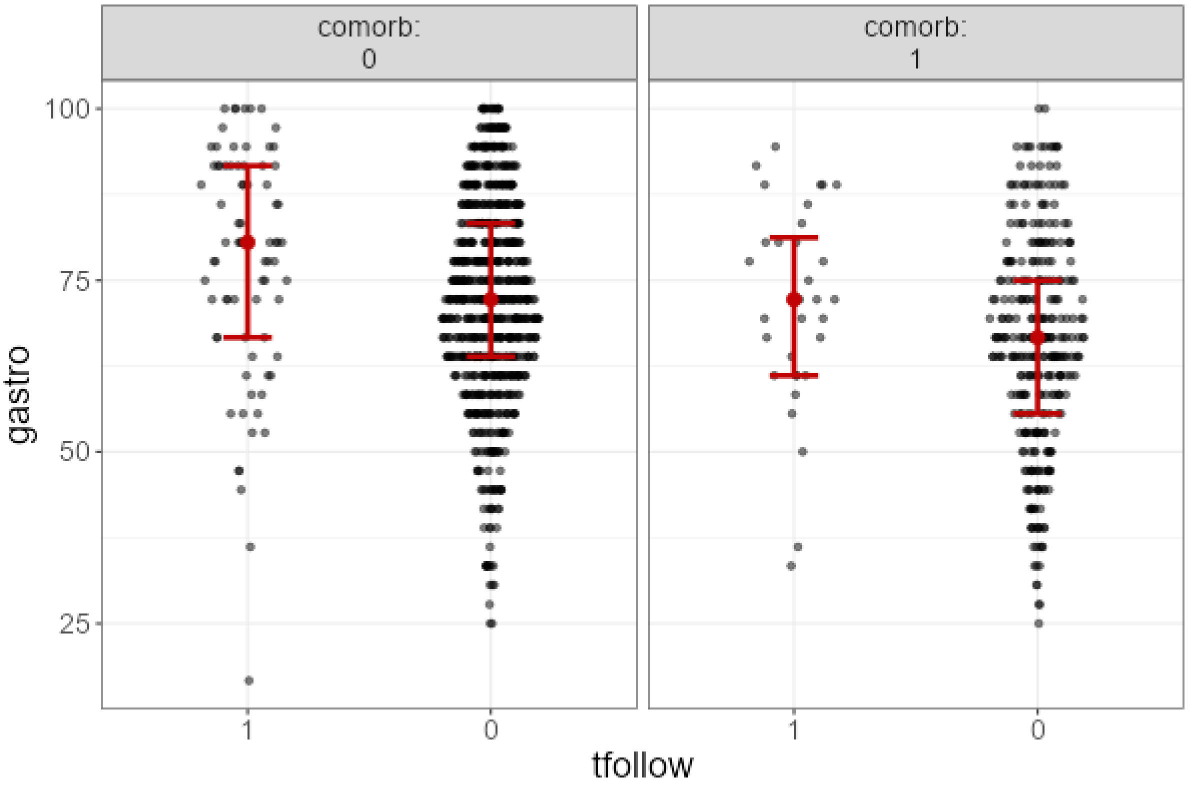

Mixed Model

Model Info

Info	
Estimate	Linear mixed model fit by REML
Call	qol ~ 1 + comorb + treatment + tfollow + comorb:treatment + comorb:tfollow+(1 | id)
AIC	2540.421
BIC	2555.998
LogLikel.	-1254.695
R-squared Marginal	0.207
R-squared Conditional	0.630
Converged	yes
Optimizer	bobyqa

[3]

Model Results

Fixed Effect Omnibus tests

	F	Num df	Den df	p
comorb	14.700	1	141	< .001
treatment	62.543	1	217	< .001
tfollow	0.426	1	217	0.515
comorb ✻ treatment	0.136	1	217	0.713
comorb ✻ tfollow	0.240	1	217	0.625

Note. Satterthwaite method for degrees of freedom

Fixed Effects Parameter Estimates

Names	Effect	Estimate	SE	95% Confidence Interval Lower	Upper	df	t	p
(Intercept)	(Intercept)	78.207	1.26	75.74	80.68	216	62.096	< .001
comorb1	1 - 0	-7.990	2.32	-12.53	-3.45	215	-3.451	< .001
treatment1	1 - 0	9.466	1.25	7.02	11.91	217	7.594	< .001
tfollow1	1 - 0	0.190	1.27	-2.30	2.68	218	0.150	0.881
comorb1 ✻ treatment1	1 - 0 ✻ 1 - 0	-0.844	2.29	-5.33	3.64	217	-0.369	0.713
comorb1 ✻ tfollow1	1 - 0 ✻ 1 - 0	1.146	2.34	-3.44	5.73	217	0.490	0.625

Random Components

Groups	Name	SD	Variance	ICC
id	(Intercept)	8.39	70.4	0.533
Residual		7.85	61.7	

Note. Number of Obs: 339 , groups: id 119

Simple Effects

Simple effects of treatment : Omnibus Tests

Moderator levels: comorb	F	Num df	Den df	p
0	57.7	1.00	217	< .001
1	20.2	1.00	217	< .001

Simple effects of treatment : Parameter estimates

Moderator levels: comorb	contrast	Estimate	SE	95% Confidence Interval Lower	Upper	df	t	p
0	1 - 0	9.47	1.25	7.01	11.9	217	7.59	< .001
1	1 - 0	8.62	1.92	4.84	12.4	217	4.50	< .001

Note. Simple effects are estimated keeping constant other independent variable(s) in the model

Effects Plots

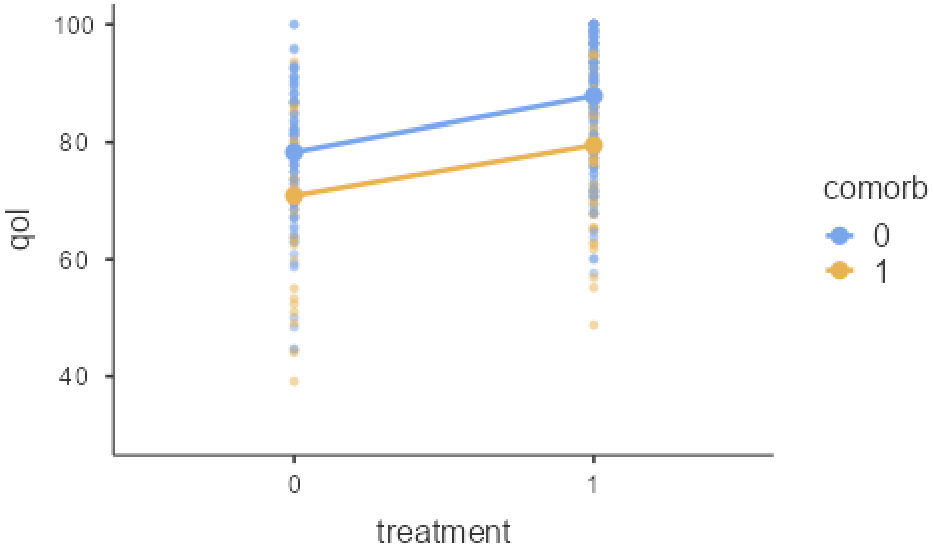

Mixed Model

Model Info

Info	
Estimate	Linear mixed model fit by REML
Call	dep ~ 1 + comorb + treatment + tfollow + comorb:treatment + comorb:tfollow+(1 | id)
AIC	1466.864
BIC	1501.356
LogLikel.	-727.386
R-squared Marginal	0.133
R-squared Conditional	0.676
Converged	yes
Optimizer	bobyqa

[3]

Model Results

Fixed Effect Omnibus tests

	F	Num df	Den df	p
comorb	14.648	1	137	< .001
treatment	8.394	1	218	0.004
tfollow	1.974	1	218	0.161
comorb ✻ treatment	0.919	1	218	0.339
comorb ✻ tfollow	2.309	1	218	0.130

Note. Satterthwaite method for degrees of freedom

Fixed Effects Parameter Estimates

Names	Effect	Estimate	SE	95% Confidence Interval Lower	95% Confidence Interval Upper	df	t	p
(Intercept)	(Intercept)	2.3052	0.275	1.765	2.845	193	8.368	< .001
comorb1	1 - 0	1.8948	0.507	0.902	2.888	192	3.740	< .001
treatment1	1 - 0	-0.8640	0.244	-1.343	-0.385	218	-3.538	< .001
tfollow1	1 - 0	0.0263	0.250	-0.464	0.516	219	0.105	0.916
comorb1 ✻ treatment1	1 - 0 ✻ 1 - 0	0.4297	0.448	-0.449	1.308	218	0.959	0.339
comorb1 ✻ tfollow1	1 - 0 ✻ 1 - 0	-0.6972	0.459	-1.597	0.202	218	-1.520	0.130

Random Components

Groups	Name	SD	Variance	ICC
id	(Intercept)	1.99	3.97	0.627
Residual		1.54	2.36	

Note. Number of Obs: 338 , groups: id 119

Simple Effects

Simple effects of treatment : Omnibus Tests

Moderator levels: comorb	F	Num df	Den df	p
0	12.52	1.00	218	< .001
1	1.34	1.00	218	0.249

Simple effects of treatment : Parameter estimates

Moderator levels: comorb	contrast	Estimate	SE	95% Confidence Interval Lower	95% Confidence Interval Upper	df	t	p
0	1 - 0	-0.864	0.244	-1.35	-0.383	218	-3.54	< .001
1	1 - 0	-0.434	0.376	-1.17	0.306	218	-1.16	0.249

Note. Simple effects are estimated keeping constant other independent variable(s) in the model

Effects Plots

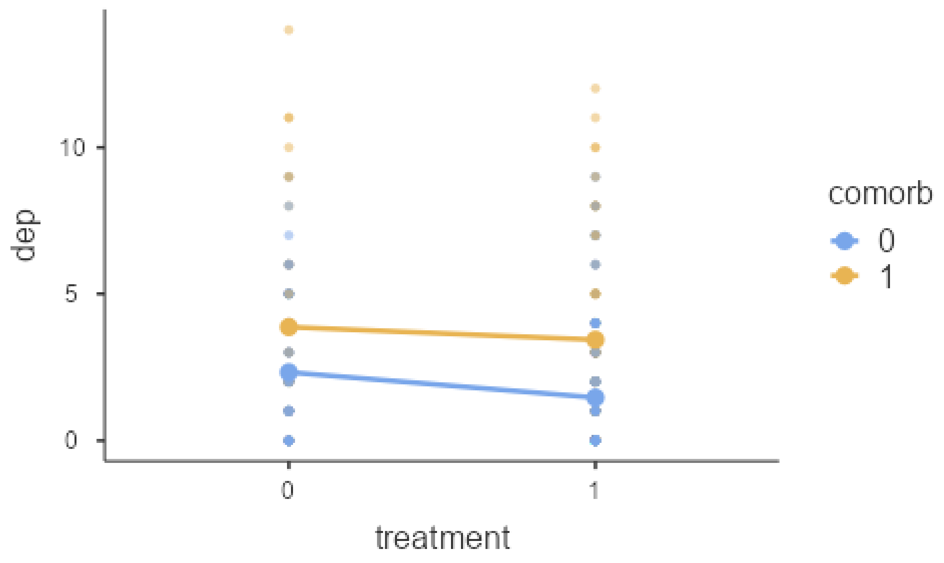

Mixed Model

Model Info

Info	
Estimate	Linear mixed model fit by REML
Call	faces ~ 1 + comorb + treatment + tfollow + comorb:treatment + comorb:tfollow+(1 | id)
AIC	1551.173
BIC	1584.236
LogLikel.	-768.814
R-squared Marginal	0.152
R-squared Conditional	0.489
Converged	yes
Optimizer	bobyqa

[3]

Model Results

Fixed Effect Omnibus tests

	F	Num df	Den df	p
comorb	7.912	1	158	0.006
treatment	21.208	1	222	< .001
tfollow	8.599	1	222	0.004
comorb ✻ treatment	8.39e-4	1	222	0.977
comorb ✻ tfollow	0.572	1	222	0.450

Note. Satterthwaite method for degrees of freedom

Fixed Effects Parameter Estimates

				95% Confidence Interval				
Names	Effect	Estimate	SE	Lower	Upper	df	t	p
(Intercept)	(Intercept)	5.7764	0.276	5.235	6.317	259	20.9254	< .001
comorb1	1 - 0	0.9950	0.507	9.37e-4	1.989	258	1.9618	0.051
treatment1	1 - 0	-1.3001	0.310	-1.907	-0.693	222	-4.1985	< .001
tfollow1	1 - 0	-1.0714	0.315	-1.689	-0.453	223	-3.3982	< .001
comorb1 ✻ treatment1	1 - 0 ✻ 1 - 0	-0.0165	0.568	-1.130	1.097	222	-0.0290	0.977
comorb1 ✻ tfollow1	1 - 0 ✻ 1 - 0	0.4392	0.581	-0.699	1.578	222	0.7561	0.450

Random Components

Groups	Name	SD	Variance	ICC
id	(Intercept)	1.59	2.52	0.398
Residual		1.95	3.82	

Note. Number of Obs: 339 , groups: id 119

Simple Effects

Simple effects of treatment : Omnibus Tests

Moderator levels				
comorb	F	Num df	Den df	p
0	17.63	1.00	222	< .001
1	7.64	1.00	222	0.006

Simple effects of treatment : Parameter estimates

Moderator levels				95% Confidence Interval				
comorb	contrast	Estimate	SE	Lower	Upper	df	t	p
0	1 - 0	-1.30	0.310	-1.91	-0.690	222	-4.20	< .001
1	1 - 0	-1.32	0.476	-2.26	-0.378	222	-2.76	0.006

Note. Simple effects are estimated keeping constant other independent variable(s) in the model

Effects Plots

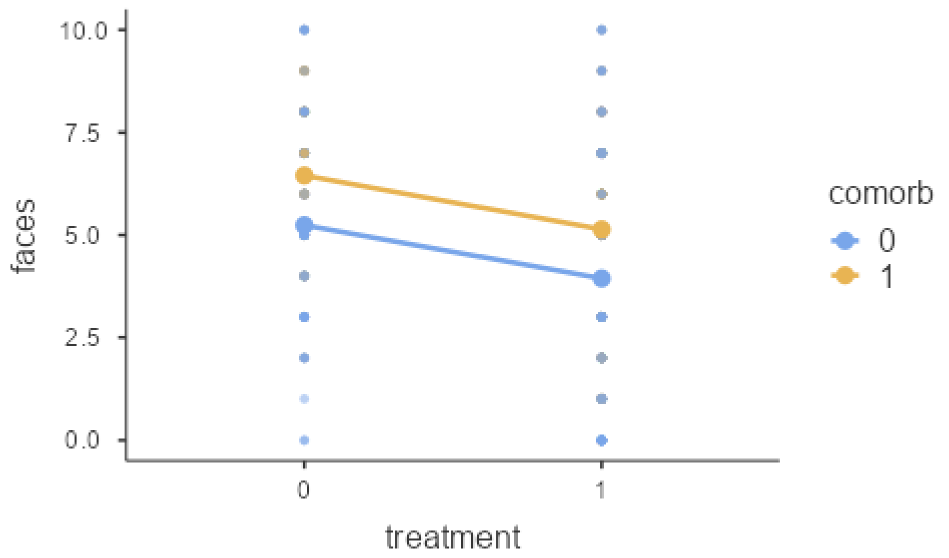

Mixed Model

Model Info

Info	
Estimate	Linear mixed model fit by REML
Call	scas ~ 1 + comorb + treatment + tfollow + comorb:treatment + comorb:tfollow+(1 | id)
AIC	2137.533
BIC	2160.137
LogLikel.	-1056.776
R-squared Marginal	0.171
R-squared Conditional	0.736
Converged	yes
Optimizer	bobyqa

[3]

Model Results

Fixed Effect Omnibus tests

	F	Num df	Den df	p
comorb	20.8081	1	132	< .001
treatment	13.6357	1	217	< .001
tfollow	0.0232	1	217	0.879
comorb ✻ treatment	3.1243	1	217	0.079
comorb ✻ tfollow	0.4281	1	217	0.514

Note. Satterthwaite method for degrees of freedom

Fixed Effects Parameter Estimates

Names	Effect	Estimate	SE	95% Confidence Interval Lower	Upper	df	t	p
(Intercept)	(Intercept)	11.460	0.776	9.938	12.98	179	14.764	< .001
comorb1	1 - 0	5.369	1.428	2.570	8.17	177	3.760	< .001
treatment1	1 - 0	-3.184	0.635	-4.429	-1.94	217	-5.010	< .001
tfollow1	1 - 0	0.300	0.651	-0.975	1.57	218	0.461	0.646
comorb1 ✻ treatment1	1 - 0 ✻ 1 - 0	2.061	1.166	-0.224	4.35	217	1.768	0.079
comorb1 ✻ tfollow1	1 - 0 ✻ 1 - 0	-0.781	1.194	-3.122	1.56	217	-0.654	0.514

Random Components

Groups	Name	SD	Variance	ICC
id	(Intercept)	5.86	34.3	0.682
Residual		4.00	16.0	

Note. Number of Obs: 338 , groups: id 119

Simple Effects

Simple effects of treatment : Omnibus Tests

Moderator levels comorb	F	Num df	Den df	p
0	25.10	1.00	217	< .001
1	1.32	1.00	217	0.252

Simple effects of treatment : Parameter estimates

Moderator levels comorb	contrast	Estimate	SE	95% Confidence Interval Lower	Upper	df	t	p
0	1 - 0	-3.18	0.635	-4.44	-1.931	217	-5.01	< .001
1	1 - 0	-1.12	0.978	-3.05	0.805	217	-1.15	0.252

Note. Simple effects are estimated keeping constant other independent variable(s) in the model

Effects Plots

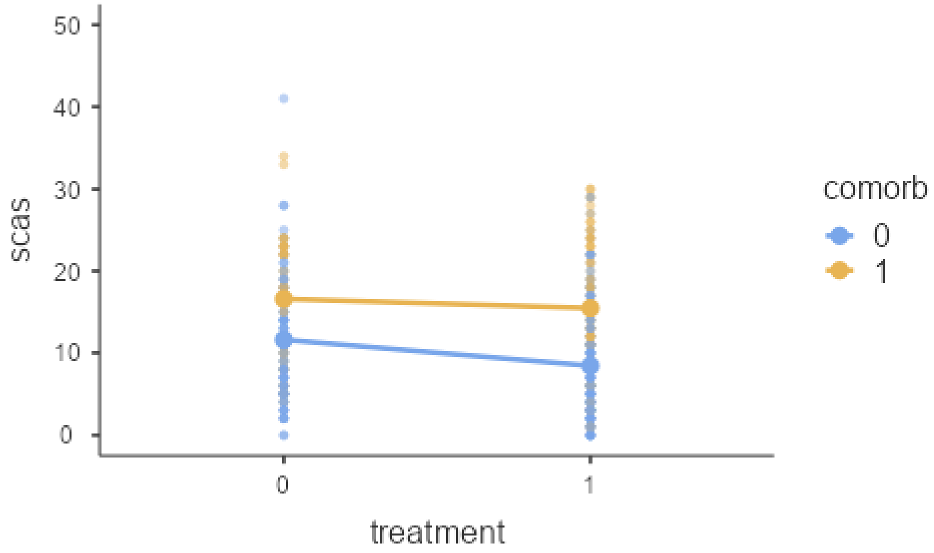

Mixed Model

Model Info

Info	
Estimate	Linear mixed model fit by REML
Call	vsi ~ 1 + comorb + treatment + tfollow + comorb:treatment + comorb:tfollow+(1 | id)
AIC	2127.195
BIC	2149.924
LogLikel.	-1051.670
R-squared Marginal	0.250
R-squared Conditional	0.579
Converged	yes
Optimizer	bobyqa

[3]

Model Results

Fixed Effect Omnibus tests

	F	Num df	Den df	p
comorb	12.4186	1	149	< .001
treatment	68.7343	1	216	< .001
tfollow	1.2721	1	217	0.261
comorb ✻ treatment	1.6438	1	216	0.201
comorb ✻ tfollow	0.0636	1	217	0.801

Note. Satterthwaite method for degrees of freedom

Fixed Effects Parameter Estimates

Names	Effect	Estimate	SE	95% Confidence Interval		df	t	p
				Lower	Upper			
(Intercept)	(Intercept)	10.686	0.661	9.390	11.983	243	16.156	< .001
comorb1	1 - 0	2.999	1.215	0.617	5.382	242	2.468	0.014
treatment1	1 - 0	-6.296	0.717	-7.701	-4.891	216	-8.783	< .001
tfollow1	1 - 0	-0.590	0.733	-2.027	0.847	218	-0.804	0.422
comorb1 ✻ treatment1	1 - 0 ✻ 1 - 0	1.687	1.315	-0.892	4.265	216	1.282	0.201
comorb1 ✻ tfollow1	1 - 0 ✻ 1 - 0	-0.339	1.347	-2.979	2.300	217	-0.252	0.801

Random Components

Groups	Name	SD	Variance	ICC
id	(Intercept)	3.99	16.0	0.438
Residual		4.52	20.4	

Note. Number of Obs: 338 , groups: id 119

Simple Effects

Simple effects of treatment : Omnibus Tests

Moderator levels				
comorb	F	Num df	Den df	p
0	77.1	1.00	216	< .001
1	17.5	1.00	216	< .001

Simple effects of treatment : Parameter estimates

Moderator levels				95% Confidence Interval				
comorb	contrast	Estimate	SE	Lower	Upper	df	t	p
0	1 - 0	-6.30	0.717	-7.71	-4.88	216	-8.78	< .001
1	1 - 0	-4.61	1.103	-6.78	-2.44	216	-4.18	< .001

Note. Simple effects are estimated keeping constant other independent variable(s) in the model

Effects Plots

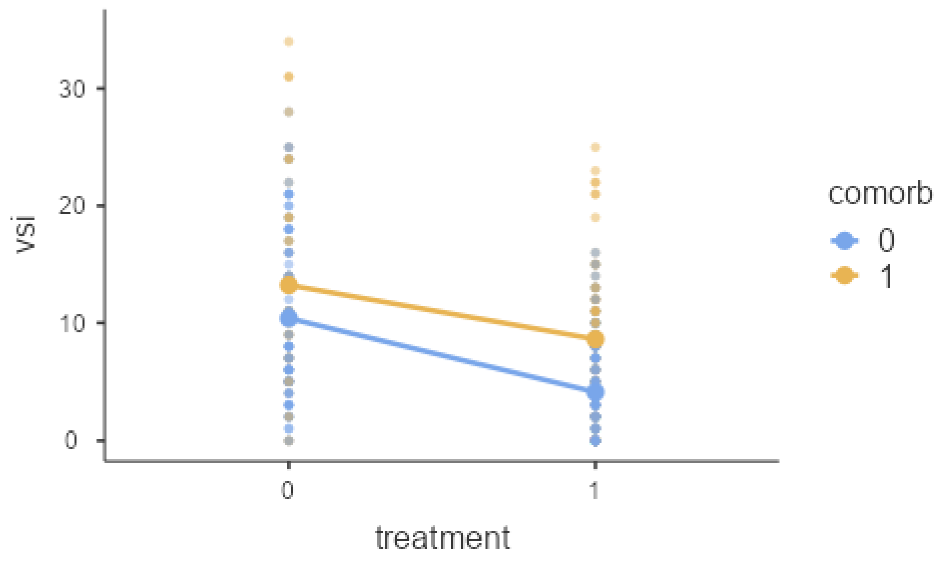

Mixed Model

Model Info

Info	
Estimate	Linear mixed model fit by REML
Call	gastro ~ 1 + comorb + treatment + tfollow + comorb:treatment + comorb:tfollow+(1 | id)
AIC	9221.1306
BIC	9246.5947
LogLikel.	-4594.8350
R-squared Marginal	0.0641
R-squared Conditional	0.6953
Converged	yes
Optimizer	bobyqa

[3]

Model Results

Fixed Effect Omnibus tests

	F	Num df	Den df	p
comorb	7.675	1	138	0.006
treatment	19.445	1	1109	< .001
tfollow	0.716	1	1110	0.398
comorb ✻ treatment	0.733	1	1109	0.392
comorb ✻ tfollow	0.583	1	1110	0.445

Note. Satterthwaite method for degrees of freedom

Fixed Effects Parameter Estimates

				95% Confidence Interval				
Names	Effect	Estimate	SE	Lower	Upper	df	t	p
(Intercept)	(Intercept)	72.155	1.43	69.35	74.96	119	50.5007	< .001
comorb1	1 - 0	-7.736	2.63	-12.89	-2.58	119	-2.9413	0.004
treatment1	1 - 0	5.104	1.06	3.03	7.17	1110	4.8337	< .001
tfollow1	1 - 0	0.108	1.42	-2.67	2.88	1112	0.0760	0.939
comorb1 ✻ treatment1	1 - 0 ✻ 1 - 0	-1.659	1.94	-5.46	2.14	1109	-0.8560	0.392
comorb1 ✻ tfollow1	1 - 0 ✻ 1 - 0	1.993	2.61	-3.12	7.11	1110	0.7638	0.445

Random Components

Groups	Name	SD	Variance	ICC
id	(Intercept)	12.67	160.4	0.674
Residual		8.80	77.4	

Note. Number of Obs: 1231 , groups: id 119

Simple Effects

Simple effects of tfollow : Omnibus Tests

Moderator levels				
comorb	F	Num df	Den df	p
0	0.00600	1.00	1112	0.939
1	0.91900	1.00	1109	0.338

Simple effects of tfollow : Parameter estimates

Moderator levels				95% Confidence Interval				
comorb	contrast	Estimate	SE	Lower	Upper	df	t	p
0	1 - 0	0.108	1.42	-2.67	2.89	1112	0.0760	0.939
1	1 - 0	2.100	2.19	-2.20	6.40	1109	0.9587	0.338

Note. Simple effects are estimated keeping constant other independent variable(s) in the model

Effects Plots

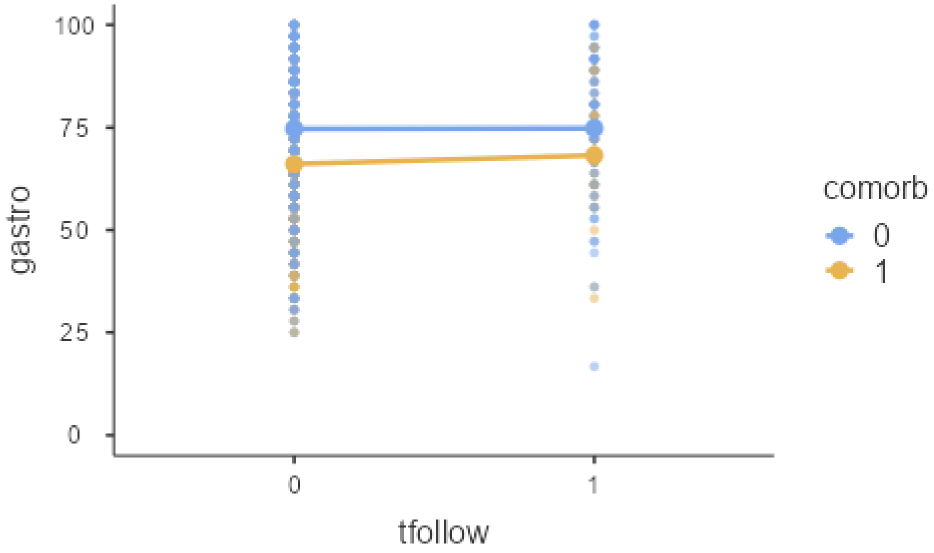

References

[1] The jamovi project (2021). *jamovi*. (Version 2.2) [Computer Software]. Retrieved from https://www.jamovi.org.

[2] R Core Team (2021). *R: A Language and environment for statistical computing*. (Version 4.0) [Computer software]. Retrieved from https://cran.r-project.org. (R packages retrieved from MRAN snapshot 2021-04-01).

[3] Gallucci, M. (2019). *GAMLj: General analyses for linear models*. [jamovi module]. Retrieved from https://gamlj.github.io/.
